# Genomic diversity of non-typhoidal Salmonella found in patients suffering from gastroenteritis in Norfolk, UK

**DOI:** 10.1099/mgen.0.001468

**Published:** 2025-08-11

**Authors:** Steven J. Rudder, Bilal Djeghout, Ngozi Elumogo, Nicol Janecko, Gemma C. Langridge

**Affiliations:** 1 Quadram Institute Bioscience, Rosalind Franklin Rd, Norwich Research Park, Norwich NR4 7UQ, UK; 2 Centre for Microbial Interactions, Rosalind Franklin Rd, Norwich Research Park, Norwich NR4 7UQ, UK; 3 Eastern Pathology Alliance, Norfolk and Norwich University Hospital, Norwich NR4 7UY, UK

**Keywords:** gastroenteritis, genomic diversity, non-typhoidal *Salmonella*

## Abstract

*Salmonella* is a significant public health pathogen responsible for a wide spectrum of diseases, ranging from gastroenteritis to invasive non-typhoidal salmonellosis and enteric fever. Although advancements in whole-genome sequencing have improved surveillance and outbreak investigations, traditional single-colony sequencing methods overlook within-host diversity, potentially underestimating the complexity of infections. This study explores the genome-wide diversity of *Salmonella* strains recovered from stool samples of eight patients, with up to 20 isolates analysed per sample. A total of 156 *Salmonella enterica* isolates were recovered. All isolates from individual patients displayed consistent serovars and sequence types. Despite the serotype consistency, microevolution was observed in *S*. Java ST149 and *S*. Java ST43, with SNP analyses revealing higher diversity (13 and 5 SNP differences, respectively) compared to the clonal populations of other serovars. Phylogenetic analysis of *S*. Java ST149 isolates from Patient 2 revealed distinct branching, driven by mutations in genes such as *secY* and *cnoX*, while *S*. Java ST43 isolates from Patient 1 displayed multiple clades with notable SNPs affecting transcriptional regulators. Genome structure (GS) analyses using hybrid assemblies identified uniform GS1.0 across all isolates. Antimicrobial resistance (AMR) profiling revealed the presence of multidrug efflux pump genes (*mdsA* and *mdsB*) in all isolates. However, *S*. Typhimurium isolates from Patient 4 exhibited additional AMR genes, including *sul2*, *aph(3'')-Ib* and *blaTEM-1*, associated with an 8.7 kb resistance region. A single isolate from Patient 4 lacked these additional genes due to the deletion of a ~19 kb genomic region, highlighting structural variation as a driver of phenotypic differences. These findings emphasize the genetic diversity of *Salmonella* within hosts, particularly in serovars such as *S.* Java, and underscore the limitations of single-colony sequencing in capturing this complexity. The study highlights the utility of hybrid sequencing strategies for comprehensive analysis of genome variation, offering valuable insights into transmission dynamics, AMR and evolutionary processes in *Salmonella*.

**Impact Statement**

This study provides a transformative perspective on *Salmonella* genomics, uncovering the significant within-host diversity of *Salmonella* populations through the analysis of multiple single-colony isolates. By leveraging hybrid sequencing technologies, we reveal critical insights into the microevolution of *Salmonella* during infection, capturing genome-wide variation at both single-nucleotide and chromosomal scales. Our findings highlight the inherent limitations of traditional single-colony sequencing in detecting within-host diversity, particularly in genetically diverse serovars such as *S.* Java ST149.

The identification of antimicrobial resistance determinants and structural genomic variation demonstrates the dynamic nature of *Salmonella* genomes, offering crucial implications for clinical management and public health surveillance. Importantly, we show that within-host diversity may influence epidemiological interpretations, particularly in outbreak investigations and source attribution, where genetic differences between isolates can mask deeper transmission links.

## Data Summary

All relevant supporting data are available in the accompanying supplementary data files. The online version of this article contains five supplementary documents.

All *Salmonella* isolate genome sequences are available in the National Center for Biotechnology Information Sequence Read Archive (SRA) under the BioProject accession number PRJNA1230128. SRA accession numbers and associated metadata for isolate genomes are included in the Supplementary Material 1 (available in the online Supplementary Material).

## Introduction

*Salmonella* is a significant public health pathogen, causing a spectrum of diseases ranging from gastroenteritis to enteric fever and invasive non-typhoidal salmonellosis [1]. Phylogenetic analysis of the *Salmonella* genus identifies three species (*Salmonella bongori*, *Salmonella arizonae* and *Salmonella enterica*), with *S. enterica* comprising ten subspecies: *enterica*, *salamae*, *arizonae*, *diarizonae*, *houtenae*, *indica*, *londinensis*, *brasiliensis*, *hibernicus*, *essexiensis* and *reptilium* [2]. Within *S. enterica* subsp. *enterica*, over 1,500 serovars have been described based on antigenic formulae, and further differentiation is commonly achieved through MLST to assign sequence types (STs) [3]. Enteric fever is caused by the *S. enterica* subsp. *enterica* serovars Typhi and Paratyphi A, B and C [4,5], while non-typhoidal invasive diseases are often associated with *S. enterica* subsp. *enterica* serovars Choleraesuis, Dublin, Panama, Virchow and Typhimurium ST 313 [6,8]. In contrast, gastroenteritis can be caused by a wide range of non-typhoidal *Salmonella* (NTS), not limited to *S. enterica* subsp. *enterica* [2]. For example, *S. enterica* subsp. *salamae* has been recorded as a rare cause of human infections in the UK [9]. A separate distinction is made for *S. enterica* subsp. *enterica* serovar Paratyphi B variant Java, a monophasic variant of *S. enterica* subsp. *enterica* serovar Paratyphi B which is classified as NTS due to its broad host range and association with gastroenteritis rather than enteric fever [10].

Symptoms of diarrhoea, mild fever and stomach cramps are characteristic of gastroenteritis caused by *Salmonella*, termed salmonellosis. Some patients also experience nausea, vomiting, headaches and muscle pains [11]. In the UK, most cases are described as mild with symptoms lasting 4–7 days [12]. Individuals with prolonged symptoms, as well as at-risk groups such as the elderly, young children and immunocompromised individuals, may require medical intervention to manage the infection, particularly due to the risks posed by invasive strains capable of causing bacteraemia [13].

Infectious dose estimates vary according to serovar, health, age and the vehicle of transmission. Generally, the infectious dose is considered to be between 10^3^ and 10^5^ viable bacterial cells [14,15]. Common sources of *Salmonella* include poultry, meat, eggs and egg products, dairy products, processed foods and contaminated water [16,17]. Other sources of note are pets such as reptiles, amphibians and dogs, as well as pet food [16,18].

In the UK, *Salmonella*-induced gastroenteritis causes an estimated 38,000 community cases annually [19]. Each year, an average of 8,000 cases of salmonellosis are reported in England and Wales through general practitioners (GPs) and hospital inpatients [20,21]. In 2022, the UK Health Security Agency (UKHSA) documented 11 *Salmonella* outbreaks in England, affecting 591 people and resulting in 3 deaths, with food sources identified in 10 of the outbreaks [21]. During the same time period, the European Union reported 1,014 *Salmonella* outbreaks, with 65,208 cases and 81 deaths. Among these, 200 cases were classified as having strong evidence of source attribution, with 151 outbreaks linked to specific food vehicles [22]. Tracing outbreaks to their source is essential for controlling the spread of infection; however, this task is hindered by the genetic diversity of *Salmonella* and the intricate nature of global food supply chains [23,24].

High-throughput short-read whole-genome sequencing (WGS) technologies have enabled a transition from biochemical-based typing methods to analysis of DNA sequences [25]. The reliability and high resolution of DNA sequence-based analysis have prompted public health agencies around the world to adopt DNA sequencing as their gold standard method for surveillance and outbreak investigations [25,31].

The standard protocols for WGS analysis involve selecting a single colony from a culture plate as input material for DNA extraction and sequencing [31,33]. While this approach provides an accurate representation of a single *Salmonella* genome, within-patient *Salmonella* diversity cannot be assessed. This is particularly important in cases where multiple *Salmonella* strains may coexist within a patient or an outbreak. This convention creates a gap in our knowledge of *Salmonella* diversity within a patient.

Recent studies have identified genome-level diversity within a single-host infection for various human pathogens, including *Burkholderia dolosa* [34], *Campylobacter* [35], *Clostridium difficile* [36], *Helicobacter pylori* [37], *Mycobacterium tuberculosis* [38], *Staphylococcus aureus* [39,40] and *Streptococcus pneumoniae* [41]. If a patient is infected with multiple strains, STs or a population containing significant SNPs, our ability to effectively conduct surveillance and accurately reconstruct transmission chains from a single colony is compromised.

Among the numerous STs of *S. enterica*, certain lineages exhibit marked differences in genetic diversity, reflecting their evolutionary histories, ecological niches and host ranges [42]. For instance, *S. enterica* subsp. *enterica* serovar Enteritidis ST11 is highly clonal and globally distributed, driven by its association with poultry and widespread dissemination through the food industry [43,45]. Similarly, *S. enterica* subsp. *enterica* serovar Paratyphi B variant Java ST43, often linked to aquaculture and livestock, is relatively clonal due to selective pressures such as antimicrobial use [46]. In contrast, *S. enterica* subsp. *enterica* serovar Typhimurium ST34 demonstrates significant genetic diversity, fuelled by its broad host range, environmental adaptability and frequent acquisition of mobile genetic elements, including antimicrobial resistance (AMR) genes [47,48]. Lesser-known STs, such as *S. enterica* subsp. *enterica* serovars Paratyphi B variant Java ST149 and Anatum ST5197, remain poorly characterized, potentially representing more diverse groups due to less-defined ecological niches. Beyond *S. enterica* subsp. *enterica*, subspecies such as *S. enterica* subsp. *salamae* also display substantial genetic diversity, underscoring the potential for other subspecies to rival the diversity seen in *S. enterica* subsp. *enterica* [2,49]. These variations in genetic diversity underscore the complexity of *Salmonella* populations and highlight the importance of genomic tools for accurate characterization and surveillance.

An important source of variation present in bacterial genomes is genome structure (GS). GS variants occur when large genome fragments rearrange around repeat sequences such as ribosomal operons, insertion sequence (IS) elements, transposases, duplicated genes and/or prophages [50]. Alteration of the order and/or orientation of fragments leads to many structural variants of the same genome sequence. In the case of *Salmonella*, the genome typically organizes around the seven ribosomal operons. To enable a numbering system for comparing structural variations, the structural analysis program Socru set a baseline of GS1.0 for *Salmonella* using the genome organization of *S. enterica* subsp. *enterica* serovar Typhimurium strain LT2. Variation in GS in *Salmonella* has been shown to affect growth phenotype and gene expression [51]. The identification of GS has been significantly enhanced by long-read sequencing platforms from Oxford Nanopore and Pacific Biosciences. Hybrid sequencing strategies now enable the assessment of genome variation at both the single-nucleotide level and the chromosomal scale [51,52].

In this study, we aim to investigate the genome-level diversity of *Salmonella* strains collected from stool samples of eight individual patients, capturing within-host genomic variation through the analysis of up to 20 isolates per sample.

## Methods

### Stool collection

This study was conducted under the ethics approval of the University of East Anglia Research Ethics Committee (Ref. 2018/19-159). Human tissue (stool) research was conducted under Norwich Biorepository licence NRES number 19/EE/0089 and IRAS Project ID 259062, approved by the UK’s Human Tissue Authority. The National Health Service Eastern Pathology Alliance (EPA) network diagnostic laboratory in Norwich, UK, was the sole participating diagnostic laboratory. *Salmonella* spp. were initially identified from the stool specimens by the diagnostic laboratory using a rapid automated PCR-based culture-independent testing panel (Gastro Panel 2, EntericBio, Serosep, UK). Once *Salmonella*-positive PCR results were confirmed, the diagnostic laboratory conducted reflexive culture according to their standard operating procedures. Eight surplus diarrhoeal stool specimens were collected from the EPA diagnostic laboratory representing eight unique anonymized patients with gastroenteritis symptoms who submitted specimens to the laboratory between March 2020 and August 2022. For this study, a 5 ml aliquot of stool was placed into a sterile specimen container, transported to the Quadram Institute Bioscience and subjected to culture-based isolation within 5 days of stool specimen submission to the EPA.

### Bacterial isolation

Stool specimens were cultured for *Salmonella* using two rounds of plating on selective media. A 10 µl aliquot of each stool specimen was directly plated to bi-plates, one half containing Xylose Lysine Deoxycholate (XLD) agar (Oxoid, UK) and the other half containing Brilliance^™^ *Salmonella* Agar (BSA; Oxoid, UK). Incubation was at 37 °C for 24 h for all steps. An upper target of up to 20 colonies exhibiting typical *Salmonella* morphology (black-centred colonies on XLD or purple colonies on BSA) was selected from either agar and transferred to MacConkey agar (Oxoid, UK) for purification. As a final purification step, putative *Salmonella* isolates were cultured onto Tryptic Soy Agar plates (Oxoid, UK). Isolates were preserved in Brucella broth supplemented with 17.5% glycerol.

### DNA extraction

DNA extraction was carried out using the Fire Monkey HMW DNA extraction kit (RevoluGen, Hadfield, UK) in 96-well format utilizing the positive air pressure on the Resolvex A200 robotic platform (Tecan) using an adapted method: STET1 (8% sucrose, 50 mM Tris-HCl, 50 mM EDTA, 5% Triton X-100) buffer was utilized with 30 mg ml^−1^ lysozyme during the lysis step. The DNA was quantified using Quant-IT broad range (Thermo Scientific, Paisley, UK) with a GloMax^®^ Discover Microplate Reader (Promega, Southampton, UK). A subset of samples was analysed with FemtoPulse (Agilent Technologies, Wokingham, UK) to confirm the presence of high molecular weight DNA.

### Sequencing

Genomic DNA was normalized to 5 ng µl^−1^ in EB buffer (10 mM Tris-HCl). For tagmentation, a master mix of 0.5 µl Tagmentation Buffer (TB1), 0.5 µl Bead-Linked Transposomes (Illumina, Cat. No. 20018704) and 4 µl PCR-grade water was prepared, and 5 µl was added to each well of a 96-well plate. Then, 2 µl of normalized DNA (10 ng total) was added, mixed and incubated at 55 °C for 15 min in a PCR block. A PCR master mix was prepared with 10 µl KAPA 2G Fast HotStart ReadyMix (Merck, Cat. No. KK5601) and 2 µl PCR-grade water per reaction. To each well, 12 µl of this mix and 1 µl of a 10 µM Illumina primer mix (containing both P5 and P7 9 bp barcodes) were added. Finally, 7 µl of the tagmented DNA was added. PCR was performed with an initial incubation at 72 °C for 3 min, denaturation at 95 °C for 1 min, followed by 14 cycles of 95 °C for 10 s, 55 °C for 20 s and 72 °C for 3 min.

Libraries were quantified using the Promega QuantiFluor^®^ dsDNA System (Cat. No. E2670) on a GloMax^®^ Discover Microplate Reader, pooled in equimolar amounts and size-selected using double-SPRI with 0.5× and 0.7×bead volumes [Illumina DNA Prep (M) Tagmentation, 96 samples, Cat. No. 20060059]. The final pool was quantified using a Qubit 3.0 and assessed on a D5000 ScreenTape (Agilent, Cat. No. 5067-5579) with the TapeStation 4200 to determine molarity. Sequencing was performed on an Illumina NextSeq 500 using a Mid Output 300-cycle flowcell (Cat. No. FC-404-2003), loaded at 1.5 pM with a 1% PhiX spike-in (PhiX Control v3, Cat. No. FC-110-3001), following Illumina’s recommended denaturation and loading protocol. For long-read sequencing, isolate DNA was sequenced in batches of 48 on a MinION using R9.4.1 flow cells (FLO-MIN106) in conjunction with Ligation Sequencing Kit (SQK-LSK109) and Native Barcoding Expansion 96 kit (EXP-NBD196) [Oxford Nanopore Technologies (ONT), Oxford, UK]. Flow cells were run for 72 h. Raw sequencing data were collected using ONT MinKNOW software (v4.0.5) and subjected to local base-calling, de-multiplexing and barcode trimming using ONT Guppy (v5.0.11). Short-read preprocessor fastp (v0.19.5) was used to remove adaptor and low-quality sequences from the Illumina reads. A minimum Illumina read coverage of 60× was required for a within-group reference isolate, and a minimum of 40× was required for an isolate to be included in the SNP analysis. No minimum coverage requirements were set for the ONT dataset. Isolates were resequenced if an isolate failed to yield a complete genome orientation data as reported by Socru (Galaxy Version 2.2.4); this was used as a proxy for a complete circular genome. Illumina and ONT datasets were typed using SeqSero2 (Galaxy Version 1.2.1) to ensure that files were matches. Samples failing to meet these requirements were removed from the study.

### Hybrid genome assembly

Short reads were quality-controlled using fastp and visualized using MultiQC (v1.11) [53,54]. Long-read sequences were filtered for high quality using Filtlong (v0.2.0) [55] with Illumina short-read sets used as external reference with Min. length=1,000, Min. mean quality=10 and Trim non-k-mer-matching activated. Hybrid assembly was run using Unicycler (v0.4.8.0) [56]. The NCBI prokaryotic genome annotation pipeline was used to annotate genomes with *Salmonella* set as the genus [57].

### *In silico* subtyping, AMR predictions and variation analysis

Sequence analysis was performed on the open-source Galaxy platform [58]. *In silico* typing was carried out using SeqSero2 (Galaxy Version 1.2.1) [59] and MLST (Galaxy Version 2.16.1) [60]. AMR genes were predicted using abriTAMR (Galaxy Version 1.0.14) (DOI 10.5281/zenodo.7370627). The order and orientation of each chromosome were analysed using *socru* (Galaxy Version 2.2.4) [50] or by manual alignment and visualization in Artemis Comparison Tool (v18.0.2) [61]. Socru identifies the order and orientation of rRNA operons to detect large-scale genome rearrangements. Operon patterns are compared against species-specific references included in Socru’s built-in database. SNP analysis was carried out using snippy4/snippy-core (Galaxy Version 4.4.3, https://github.com/tseemann/snippy) mapping fastp-processed short reads using the default settings (mapping quality=60, minimum coverage=10, minimum fraction=0.9 and minimum quality=100) to a hybrid assembly reference selected from each patient. IQ-tree (Galaxy Version 2.1.2) [62,63] was used for tree construction using the ultrafast bootstrap default method, 1,000 iterations and a minimum correlation coefficient of 0.99. For each patient, one hybrid assembly was selected to be the reference based on quality metrics including Illumina read coverage ≥60×, a complete GS solved using *socru* [50], low contamination score (>0.5, approximately >0.5% of the genome is estimated to originate from contaminant sources) obtained from CheckM (Galaxy Version 1.0.11) and a CheckM strain heterogeneity of 0 [64]. Isolates meeting all criteria were therefore considered complete, uncontaminated and of reference genome quality. Paired short-read sequence data were used to align to the reference per patient. Maximum likelihood trees were visualized with iTOL (https://itol.embl.de/).

### blast search

DNA sequences of interest were identified from an annotated GenBank file within Artemis. The sequences were excised as a fasta file. A blastn search was carried out using the excised DNA sequence as input.

### Evaluating cases of *S. enterica* subsp. *salamae* in England and Wales

To evaluate the incidence of *S. enterica* subsp. *salamae* in England and Wales, a dataset consisting of 356 strains was identified using filtering parameters with EnteroBase [65]. *Salmonella* entries were filtered to include Public Health England (PHE) (UKHSA) and the GBRU (Gastrointestinal Bacteria Reference Unit) as Lab Contact and II as Subspecies. Entries with MLST information were used to form an MSTree2 embedded within EnteroBase. Entries with assembly data were downloaded and analysed using abriTAMR (Galaxy Version 1.0.1).

## Results

### Patient characterization

Stool specimens from eight patients were PCR-confirmed salmonellosis cases presenting with diarrhoea. Five cases submitted specimens to GPs, while the remaining three were submitted at Norfolk and Norwich University Hospital. Notably, two patients reported a recent travel history. The patients ranged from 2 to 77 years of age, and 7 out of 8 cases were identified as female (Supplementary Material 2).

### *Salmonella* genomic subtyping within patients

A total of 156 *Salmonella* isolates were recovered. The number of isolates per patient stool specimen ranged from 18 to 20 isolates. All isolates recovered were *S. enterica* subsp. *enterica*, except for one patient who harboured *S. enterica* subsp. *salamae* (*S. salamae*). SeqSero2 and MLST were used to obtain a serotype prediction and ST per isolate. For all isolates from each patient, a single serotype and ST was observed including two cases of *S. enterica* serovar Paratyphi B variant Java (*S*. Java) and two cases of *S. enterica* serovar Enteritidis (*S*. Enteritidis) (Table 1).

**Table 1.** Description of *Salmonella* identified in stool samples of eight gastroenteritis patients in Norfolk, UK

Patient ID	*Salmonella enterica* subsp.	Sequence type (ST)	No. of isolates per patient
Patient 1	*Salmonella enterica* subsp. *enterica* serovar Paratyphi B variant Java	43	20
Patient 2	*Salmonella enterica* subsp. *enterica* serovar Paratyphi B variant Java	149	19
Patient 3	*Salmonella enterica* subsp. *enterica* serovar Infantis	32	18
Patient 4	*Salmonella enterica* subsp. *enterica* serovar Typhimurium	34	20
Patient 5	*Salmonella enterica* subsp. *enterica* serovar Enteritidis	11	20
Patient 6	*Salmonella enterica* subsp. *salamae*	9581	20
Patient 7	*Salmonella enterica* subsp. *enterica* serovar Anatum	5197	20
Patient 8	*Salmonella enterica* subsp. *enterica* serovar Enteritidis	11	19

### Maintenance of GS

To identify if GS was uniform across the isolates obtained from each patient, the GS was solved by running *socru* on hybrid genome assemblies. Across all eight patients, the GS of isolates was uniform with no differences observed. The GS observed for all isolates was GS1.0 [50].

### AMR determinants

**Fig. 1.**
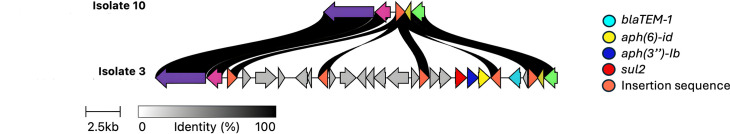
Loss of AMR-carrying transposable element in the *S*. Typhimurium genome of Patient 4. Clinker [81] schematic comparing two isolates collected from the same faecal sample from an acute case (Patient 4), with no difference in time of collection. Isolate 3 represents the consensus sequence found in 19 of 20 colonies, while isolate 10 shows a loss of a region flanked by ISs (orange) that includes four AMR genes (red, dark blue, yellow and light blue). Genes are shown as directional arrows, with identical sequences coloured consistently. Homology between the isolates is indicated by black bars; regions lacking these links are absent in isolate 10.

All isolate genomes were examined for AMR determinants using complete hybrid genome assemblies. Regardless of serovar, all genomes exhibited the presence of the *mdsA* and *mdsB* genes, which encode a multidrug resistance efflux pump [66]. For Patient 4, 19 out of 20 *S. enterica* subsp. *enterica* serovar Typhimurium (*S*. Typhimurium) isolates displayed a broader AMR determinant profile, carrying additional resistance determinants of *sul2* (sulphonamide resistance), *aph(3'')-Ib* and *aph(6)-Id* (aminoglycoside resistance) and *blaTEM-1* (beta-lactam resistance) genes. The 8.7 kb region carrying the AMR genes *sul2*, *aph(3'')-Ib*, *aph(6)-Id* and *blaTEM-1* was flanked by and contained IS15DIV transposase ISs. One *S*. Typhimurium isolate out of the 20 (isolate 10, Patient 4) contained only *mdsA* and *mdsB*. Inspection of the genome revealed a single IS at the site where *sul2*, *aph(3'')-Ib*, *aph(6)-Id* and *blaTEM-1* were expected (Fig. 1). Comparative genomics showed a ~19 kb region missing in this isolate’s genome which housed the four AMR genes alongside flagellar phase variation proteins *hin*, *fljA* and *fljB* and a PTS (phosphotransferase system) transport system. blast searches were conducted to screen for the presence of this region in other *Salmonella* and bacterial species. Two regions were queried: first, the full 22,636 bp segment flanked by the external IS15DIV elements; and second, an 8,647 bp subregion containing the four AMR genes separated by IS15DIV. Complete query coverage and 100% identity for the full IS15DIV segment were rare in the database with a total of 11 hits from three different sources, and they were all *S*. Typhimurium isolates. The smaller segment, which could be considered the AMR cassette, was much more prevalent at complete query coverage and 100% identity and included hits to *S*. Typhimurium isolates, an *Escherichia coli* isolate, a *Proteus mirabilis* isolate and two *E. coli* plasmids (accession numbers AP019762.1 and CP147536.1). blast searches are included in Supplementary Material 2.

### SNP diversity

**Fig. 2.**
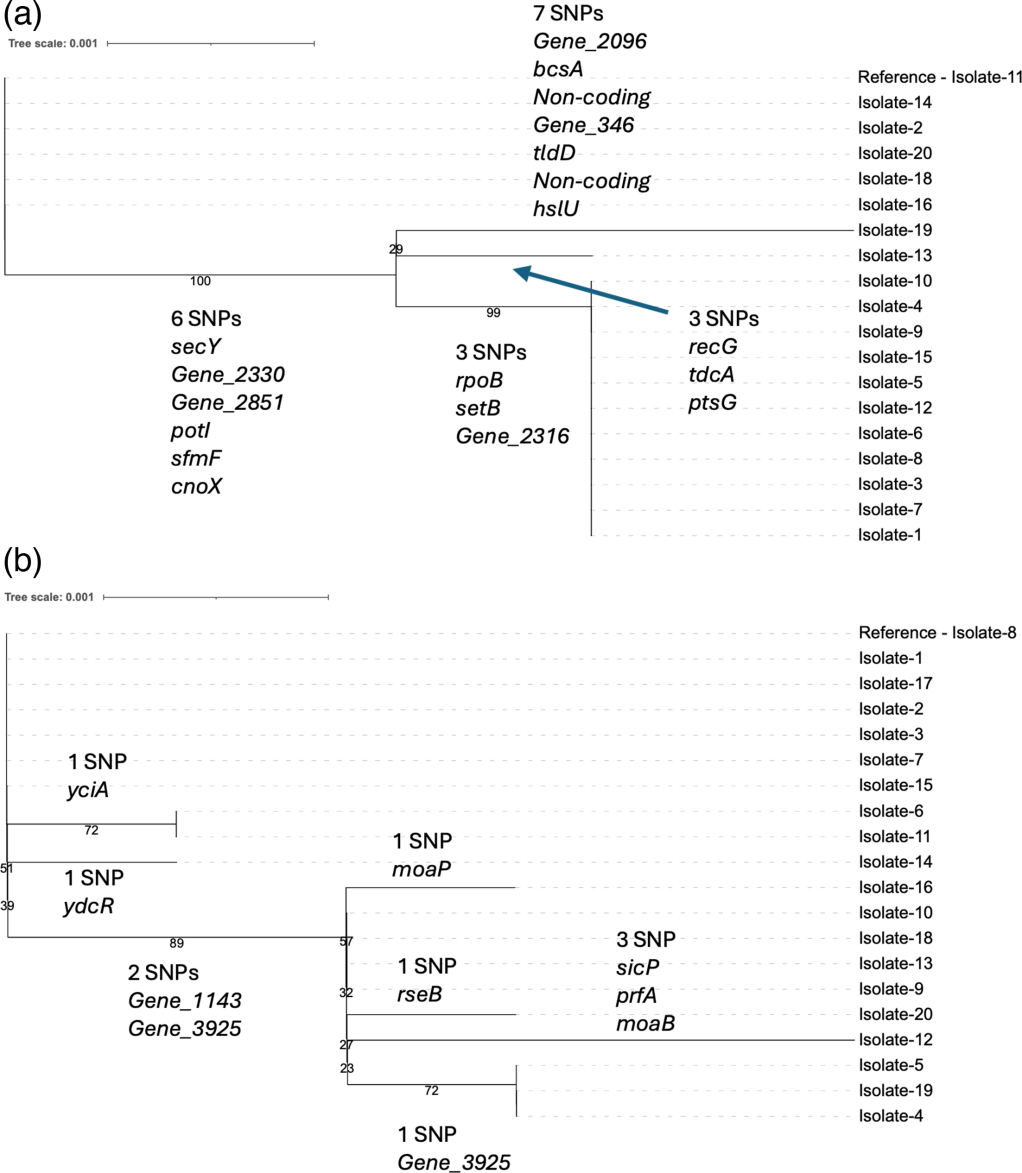
Within-patient variation in Patients 1 and 2 (*S*. Java). Tree (a): core genome maximum likelihood tree for 19 *S. enterica* subsp. *enterica* serovar Paratyphi B variant Java (*S*. Java) isolates (Patient 2). Tree (b): core genome maximum likelihood tree for 20 *S*. Java isolates (Patient 1). Tree overlaid with SNPs responsible for each branch and bootstrapping values (bootstrap replicates=1,000).

Based on the SNP analysis, all *Salmonella* isolates recovered from Patients 3 to 8 were considered clonal with a maximum SNP difference of one. *S.* Java recovered from Patients 1 and 2 was more diverse, with a maximum SNP difference of 5 and 13, respectively. The number of distinct SNPs identified per patient was as follows: Patient 1, 10 SNPs; Patient 2, 19 SNPs; Patient 3, 3 SNPs; Patient 4, 0 SNP; Patient 5, 3 SNPs; Patient 6, 0 SNP; Patient 7, 2 SNPs; and Patient 8, 1 SNP. The number of SNPs per isolate can be found in Table 2. Across the dataset, a total of 38 SNPs were observed; 26 were missense variants, 3 caused truncation by gaining a STOP codon, 2 were in non-coding DNA and 7 were synonymous Supplementary Material 1. The most diverse isolates were the *S*. Java ST149 isolates from Patient 2. The core genome phylogenetic tree revealed a process of microevolution occurring (Fig. 2). A deep branch separated a basal group of six isolates from the remaining 13. The six SNPs responsible for forming this branch were in *secY*, *potI(ydcV*), *sfmF*, *cnoX*, a hypothetical gene encoding a dimethyl sulphoxide reductase anchor subunit family protein and a hypothetical gene encoding a hydrogenase expression/formation domain-containing protein. Beyond the basal branch, three separate branches were present. Isolate 19 formed its own branch with a seven-SNP distance, isolate 13 formed its own branch with a three-SNP distance and a group of 11 isolates formed a clade at a distance of three SNPs.

**Table 2.** Number of SNPs identified in each isolate

	Isolate ID		1	2	3	4	5	6	7	8	9	10	11	12	13	14	15	16	17	18	19	20
Patient ID	*Salmonella*	Sequence type																				
Patient 1	Java	ST43	0	0	0	3	3	1	0	2	1	2	R	5	2	1	0	3	0	2	3	3
Patient 2	Java	ST149	9	0	9	9	9	9	9	9	9	9	R	9	9	0	9	0	n/a	0	13	0
Patient 3	Infantis	ST32	1	0	0	1	0	0	0	n/a	0	1	0	R	0	1	0	0	0	0	n/a	0
Patient 4	Typhimurium	ST34	0	0	R	0	0	0	0	0	0	0	0	0	0	0	0	0	0	0	0	0
Patient 5	Enteritidis	ST11	0	R	1	0	1	1	0	1	0	0	0	0	0	0	0	0	0	0	0	0
Patient 6	*salamae*	ST9581	0	0	0	0	0	0	0	0	0	0	0	0	0	0	0	0	0	0	0	R
Patient 7	Anatum	ST5197	0	0	0	0	0	1	0	0	0	0	0	0	R	0	0	0	0	1	0	0
Patient 8	Enteritidis	ST11	0	0	0	0	0	0	R	0	0	0	0	0	n/a	0	0	0	0	0	0	1

R = reference strain, n/a = no sequence data for isolate.

Three basal branches were observed in the core SNP tree for the isolates from Patient 1’s *S*. Java ST43 infection: isolate 14 appeared on its own branch, isolate 6 and Isolate 11 formed another branch and a clade of ten isolates appeared on a third branch. This clade of ten isolates was further diversified with four more branches. Isolate 4, isolate 5 and isolate 19 carried a double mutation in *Gene_3925* (Fig. 2). The gene was predicted to act as a transcriptional regulator for a PTS sugar transporter and was located upstream of the predicted PTS system subunits IIA, IIB, IIC and IID. Both SNPs in *Gene_3925* resulted in missense variants within the PTS EIIA mannose/sorbose-specific type-4 domain of the protein, a component of the enzyme II (EII) complex.

Additional mutations that appeared in multiple isolates within a single patient group included a missense variant in *bigA*, found in two *S. enterica* subsp. *enterica* serovar Infantis (*S*. Infantis) isolates from Patient 3, and a missense variant in *dnaJ*, observed in two *S*. Enteritidis isolates from Patient 5. The gene *bigA* encodes a large putative surface-exposed virulence protein associated with virulence in some intracellular pathogens [67]. The gene *dnaJ* is located within the *Salmonella* pathogenicity island II. Loss-of-function mutations in the *dnaJ* gene have been shown to increase heat resistance in *S*. Typhimurium [68]. A mutation in the metalloprotease *tldD* was identified in a single isolate from Patient 2 (Java) and Patient 5 (Enteritidis). Both mutations resulted in missense variants affecting different domains within the TldD protein. Three mutations resulting in truncated proteins were identified: in a single isolate from Patient 1 (*S*. Java), SicP was truncated from 130 amino acids to 43 amino acids; in a single isolate from Patient 5 (*S*. Enteritidis), RcnA was truncated from 284 amino acids to 104 amino acids; and in a single isolate from Patient 7 (*S. enterica* subsp. *enterica* serovar Anatum), RcsC was truncated from 948 amino acids to 642 amino acids.

### Rare case of gastroenteritis caused by *S. enterica* subsp. *salamae* in the UK

**Fig. 3.**
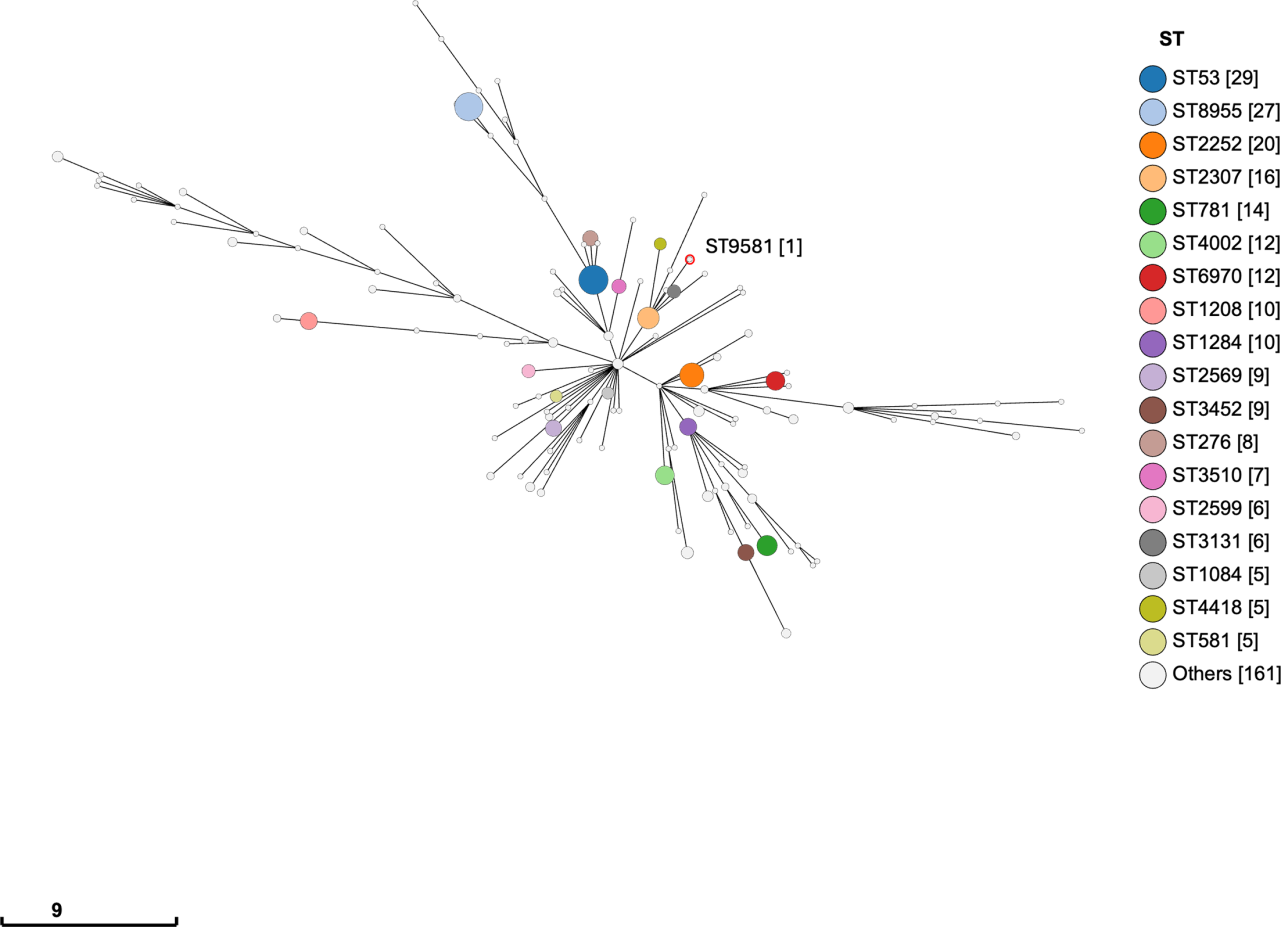
Phylogenetic relationships within *S. enterica* subsp. *salamae* from England and Wales. Minimum spanning tree (MSTree2) based on the Achtman 7-gene MLST profiles of *S. enterica* subsp. *salamae* isolates submitted to EnteroBase by the GBRU and PHE between 1956 and 2024. Each node represents a unique ST, and edges reflect the number of allelic differences across the seven housekeeping genes used in the MLST scheme. The scale bar indicates the number of loci at which alleles differ between STs (i.e. allelic distance). Patient 1’s isolate is highlighted in red and labelled with its ST and the number of occurrences: ST [occurrence].

There are 356 subspecies II (*S. enterica* subsp. *salamae*) entries in EnteroBase linked to England and Wales deposited by PHE (UKHSA) and the GBRU with MLST information. Of the 356 entries, 123 different STs were observed. The most commonly observed STs were ST53 and ST8955, with 29 and 27 entries, respectively (Fig. 3). Patient 1’s isolates were ST9581, which had a single entry. Based on MLST analysis, ST9581 was most closely related to ST2307, a serovar seen 16 times between 2015 and 2024. Analysis of the AMR profiles of 285 out of 356 genome assemblies available from EnteroBase revealed low levels of AMR determinants among subspecies II linked to gastroenteritis in the UK. The *fosA7.4* gene was observed in 27 isolates, *fosA7.2* in 1 isolate and *tet(B*) in 1 isolate. The efflux pairing *mdsA* and *mdsB* present in all of Patient 6’s *S. enterica* subsp. *salamae* (*S. salamae*) isolates was observed in 200 isolates, leaving 85 isolates with no recognizable AMR determinants.

## Discussion

To our knowledge, this is the first study analysing multiple isolates of *Salmonella* identified from individual patients’ stool samples who were suffering from gastroenteritis. This study analysed the genomic diversity of *Salmonella* within eight patients’ stool samples considering subspecies, ST, serotype, GS, AMR profile and SNPs. No mixed *Salmonella* species infections were observed, with all isolates from individual infections being identified as the same subspecies, serovar and ST. This aligns with the existing literature on *Salmonella*, where reports of multiple-serovar infections are exceptionally rare and seldom documented [69,70].

The GS of all genomes screened within a stool sample was uniform and identified as GS1.0 and is in alignment with GS1.0 being the most commonly observed GS across the *Salmonella* genus [50]. Structural deviations from GS1.0 are overrepresented in *S.* Typhi and have been linked to persistence within the human host [50,71]. Recent analysis of *S. enterica* subsp. *enterica* serovar Agona isolates from UK infections found GS1.0 to be the most dominant structure [52]. The study included isolate sequencing for acute and persistent infections within individual patients linking alterations in GS away from GS1.0 to early convalescent carriage (3 weeks–3 months). As an emerging source of genome-level variation, the impact of GS on infection severity, progression and host persistence remains unknown. To our knowledge, all patients in this study suffered from acute disease only.

Our study reveals that SNPs were detected in *Salmonella* isolates from six out of eight patients. The isolates recovered from Patient 4 (*S*. Typhimurium) and Patient 6 (*S. salamae*) had no SNP differences in a core alignment. The most diverse set of isolates was from Patient 2’s *S*. Java ST149 infection. Two clades appeared to be present in this population, separated by 9 SNPs, with isolate 19 having a 13-SNP distance from the reference. For outbreak investigations involving *Salmonella*, UKHSA uses single linkage clustering at 250, 100, 50, 25, 10, 5 and 0 SNPs, forming an SNP address [25,72]. Thresholds of zero and five SNPs are deemed to support a meaningful relatedness between isolates and their point of origin. At a threshold of ten SNPs, the relationship between isolates is less clear, although it is noted that deeper epidemiological links may be uncovered by analysis at this level, providing important clues during an outbreak investigation [72]. A key takeaway from this study is that the genome-level diversity present among Patient 2’s *S.* Java ST149 may not be accurately observed when sequencing a single isolate.

Patient 1’s *S*. Java ST43 infection formed two clades separated by two SNPs with a maximum distance from the reference of five SNPs. Three isolates carried two non-synonymous SNPs in *Gene_3925*, a sigma 54-interacting transcriptional regulator similar to the *dgaR* gene. The *dgaR* gene has previously been described as an RpoN-dependent activator of the *dgaABCDEF* operon, a mannose family PTS enabling the catabolism of d-glucosaminate to pyruvate plus glyceraldehyde-3-phosphate [73]. All PTS permeases consist of three domains, named EIIA, EIIB and EIIC, whereas mannose family PTS permeases include a fourth domain, referred to as EIID [74,75]. EII complexes are linked to the cell membrane and are specific to a particular sugar or a group of structurally similar sugars [73]. In the case of the operon led by *Gene_3925*, EIIA is a hypothetical PTS sugar transporter subunit, EIIB is annotated as a sorbose-specific EIIB component, EIIC is annotated as an *N*-acetylgalactosamine permease IIC and EIID is annotated as a PTS system mannose-specific EIID component. The dual mutation observed to *Gene_3925* is suggestive of adaptive pressure on this pathway within *S.* Java ST43 during passage through the human digestive system.

Observation of different AMR profiles for isolates from the same patient was rare. For seven of the eight patient stool samples, the *Salmonella* isolates exhibited limited AMR determinants, carrying only the efflux pump genes *mdsA* and *mdsB*. In contrast, Patient 4’s *S*. Typhimurium infection showed a notable difference in its AMR profile, with one isolate predicted to be sensitive, while the consensus profile included *sul2*, *aph(3'')-Ib*, *aph(6)-Id* and *blaTEM-1*. Long-read sequencing enabled the assembly of complete circular genomes, allowing for detailed inspection of the genome sequences of these isolates, including the location of repetitive elements and AMR determinants. Analysis revealed that these four AMR genes were located within a genomic region flanked by and containing five IS15DIV ISs (Fig. 1). IS15DIV has been associated with the co-occurrence of resistance markers in *E. coli* [76]. A blast search of the region revealed 100% homology to a plasmid from an enterohemorrhagic *E. coli* O111:H8 strain recovered from a large outbreak in Japan associated with the consumption of raw beef [77]. This transposable element has also been identified within a chromosomal drug resistance region in an ESBL-producing *S.* Typhi isolate (814995) from a UK patient with enteric fever symptoms, linked to travel history in Karachi, Pakistan, in September 2019 [78]. We hypothesize that the sensitive *S*. Typhimurium ST34 isolate lost the four AMR genes in a recombination event, leaving a single IS at this location. Observing different *Salmonella* AMR profiles within a single infection demonstrates that analysing a single colony may not accurately represent the *Salmonella* population responsible for the infection and impedes investigative conclusions. If the sensitive colony was selected as representative of the population, then valuable information about the resistance profile would have been missed. While sequencing multiple isolates individually increases the investigation power, it remains unclear how many isolates would be needed to fully capture the genetic diversity within a *Salmonella* population during an infection. For *Campylobacter*, it has been suggested that up to 80 isolates would be needed to capture 95% of core non-recombinant SNPs [35]. Using the *S*. Java isolates as a proxy, a minimum of 11 isolates would be required to detect at least one isolate with an SNP differing from the reference. To capture the full extent of within-host diversity, more than 13 isolates would be needed. This represents the first study to explore the level of genetic variation among *Salmonella* isolates from a single patient and offers an initial insight into the potential for genome-level diversity within individual infections.

A significant cost is associated with sequencing multiple isolates per patient sample. Additional costs of labour, isolating the multiple colonies, DNA extraction, DNA sequencing and data storage make a multiple-colony approach unfeasible for any large-scale diagnostic pipelines. Alternative sampling strategies such as sweep sequencing or pool-seq, where multiple isolates are DNA-extracted and sequenced as one sample, have been proposed as an alternative approach capable of capturing information at the population level [39,79, 80]. These alternative approaches would alleviate the detection limitations of using a single colony; however, both methods have their own constraints. Sweep sequencing can result in the loss of minor alleles, leading to a biassed representation of genetic diversity. Similarly, pool-seq, while capable of capturing a broader representation of population diversity, can introduce biases during pooling due to unequal DNA contributions from individual isolates, and low-frequency variants may remain undetected unless sequencing depth is sufficiently high. Furthermore, neither method provides insights into the genomic context of individual isolates, limiting their ability to resolve detailed GS and link SNPs and/or AMR profiles to individual isolates.

## Conclusion

Our sequencing of multiple *Salmonella* isolates from patient stool samples demonstrated varying levels of genome diversity within the populations of *S*. Java strains involved in gastroenteritis cases. This is informative to epidemiological investigations involving serovars or STs displaying more genetic diversity. Sequencing strategies that enable analysis of many isolates from a single patient’s sample can provide a higher resolution of genetic information, capturing the full spectrum of genetic diversity within the bacterial population. This approach can identify subtle variations and rare mutations that might otherwise go undetected. Over time, such detailed analysis could help uncover common genes under selective pressure during the infection process, shedding light on the mechanisms driving bacterial adaptation, persistence and resistance.

## Supplementary material

10.1099/mgen.0.001468 Supplementary Material 1.

10.1099/mgen.0.001468 Supplementary Material 2.

## Abbreviations

AMR: antimicrobial resistance
BSA: Brilliance™ Salmonella Agar
EII: enzyme II
EPA: Eastern Pathology Laboratory
GBRU: Gastrointestinal Bacteria Reference Unit
GP: general practitioner
GS: genome structure
IS: insertion seqeunce
NTS: non-typhoidal *Salmonella*
ONT: Oxford Nanopore Technologies
PHE: Public Health England
PTS: phosphotransferase system
SRA: Sequence Read Archive
ST: sequence type
UKHSA: UK Health Security Agency
WGS: whole-genome sequencing
XLD: Xylose Lysine Deoxycholate

## References

[1] Galán-Relaño Á, Valero Díaz A, Huerta Lorenzo B, Gómez-Gascón L, Rodríguez MAM (2023). *Salmonella* and salmonellosis: an update on public health implications and control strategies. Animals.

[2] Pearce ME, Langridge GC, Lauer AC, Grant K, Maiden MCJ (2021). An evaluation of the species and subspecies of the genus *Salmonella* with whole genome sequence data: proposal of type strains and epithets for novel *S. enterica* subspecies VII, VIII, IX, X and XI. Genomics.

[3] Urwin R, Maiden MCJ (2003). Multi-locus sequence typing: a tool for global epidemiology. Trends Microbiol.

[4] Chattaway MA, Gentle A, Nair S, Tingley L, Day M (2021). Phylogenomics and antimicrobial resistance of *Salmonella* Typhi and Paratyphi A, B and C in England, 2016-2019. Microb Genom.

[5] Marchello CS, Birkhold M, Crump JA (2020). Complications and mortality of typhoid fever: a global systematic review and meta-analysis. J Infect.

[6] Langridge GC, Wain J, Nair S (2012). Invasive salmonellosis in humans. EcoSal Plus.

[7] Ashton PM, Owen SV, Kaindama L, Rowe WPM, Lane CR (2017). Public health surveillance in the UK revolutionises our understanding of the invasive *Salmonella* Typhimurium epidemic in Africa. Genome Med.

[8] Pulford CV, Perez-Sepulveda BM, Rodwell EV, Weill FX, Baker KS (2019). *Salmonella enterica* Serovar Panama, an understudied serovar responsible for extraintestinal salmonellosis worldwide. Infect Immun.

[9] Nair S, Wain J, Connell S, de Pinna E, Peters T (2014). *Salmonella enterica* subspecies II infections in England and Wales--the use of multilocus sequence typing to assist serovar identification. J Med Microbiol.

[10] Chart H (2003). The pathogenicity of strains of *Salmonella paratyphi* B and *Salmonella java*. J Appl Microbiol.

[11] Lamichhane B, Mawad AMM, Saleh M, Kelley WG, Harrington PJ (2024). Salmonellosis: an overview of epidemiology, pathogenesis, and innovative approaches to mitigate the antimicrobial resistant infections. Antibiotics.

[12] Eng S-K, Pusparajah P, Ab Mutalib N-S, Ser H-L, Chan K-G (2015). *Salmonella*: a review on pathogenesis, epidemiology and antibiotic resistance. Front Life Sci.

[13] Katiyo S, Muller-Pebody B, Minaji M, Powell D, Johnson AP (2019). Epidemiology and outcomes of nontyphoidal *Salmonella* Bacteremias from England 2004 to 2015. J Clin Microbiol.

[14] Blaser MJ, Newman LS (1982). A review of human salmonellosis: I. Infective dose. Rev Infect Dis.

[15] Kothary MH, Babu US (2001). Infective dose of foodborne pathogens in volunteers: a review. J Food Safety.

[16] Freitas Neto O de, Penha Filho R, Barrow P, Berchieri Junior A (2010). Sources of human non-typhoid salmonellosis: a review. Braz J Poultry Sci.

[17] Pires SM, Vieira AR, Hald T, Cole D (2014). Source attribution of human salmonellosis: an overview of methods and estimates. Foodborne Pathog Dis.

[18] Mermin J, Hutwagner L, Vugia D, Shallow S, Daily P (2004). Reptiles, amphibians, and human *Salmonella* infection: a population-based, case-control study. Clin Infect Dis.

[19] Mair-Jenkins J, Borges-Stewart R, Harbour C, Cox-Rogers J, Dallman T (2017). Investigation using whole genome sequencing of a prolonged restaurant outbreak of *Salmonella* Typhimurium linked to the building drainage system, England, February 2015 to March 2016. Euro Surveill.

[20] Chattaway MA, Dallman TJ, Larkin L, Nair S, McCormick J (2019). The Transformation of Reference Microbiology Methods and Surveillance for *Salmonella* With the Use of Whole Genome Sequencing in England and Wales. Front Public Health.

[21] gov.uk (2024). Non-typhoidal *Salmonella* data 2013 to 2022. https://wwwgovuk/government/publications.

[22] European Food Safety Authority (EFSA), European Centre for Disease Prevention and Control (ECDC) (2023). The European Union One Health 2022 Zoonoses Report. EFS2.

[23] Hoffmann M, Luo Y, Monday SR, Gonzalez-Escalona N, Ottesen AR (2016). Tracing origins of the *Salmonella* Bareilly strain causing a food-borne outbreak in the United States. J Infect Dis.

[24] Bayliss SC, Locke RK, Jenkins C, Chattaway MA, Dallman TJ (2023). Rapid geographical source attribution of *Salmonella enterica* serovar Enteritidis genomes using hierarchical machine learning. eLife.

[25] Chattaway MA, Painset A, Godbole G, Gharbia S, Jenkins C (2023). Evaluation of genomic typing methods in the *Salmonella* reference laboratory in Public Health, England, 2012-2020. Pathogens.

[26] Brown E, Dessai U, McGarry S, Gerner-Smidt P (2019). Use of whole-genome sequencing for food safety and public health in the United States. Foodborne Pathog Dis.

[27] Deng X, den Bakker HC, Hendriksen RS (2016). Genomic epidemiology: whole-genome-sequencing–powered surveillance and outbreak investigation of foodborne bacterial pathogens. Annu Rev Food Sci Technol.

[28] Meumann EM, Krause VL, Baird R, Currie BJ (2022). Using genomics to understand the epidemiology of infectious diseases in the Northern Territory of Australia. *Trop Med Infect Dis*.

[29] Li W, Cui Q, Bai L, Fu P, Han H (2021). Application of whole-genome sequencing in the national molecular tracing network for foodborne disease surveillance in China. Foodborne Pathog Dis.

[30] Lee K, Izumiya H, Iyoda S, Ohnishi M, Morimoto Y (2019). Effective surveillance using multilocus variable-number tandem-repeat analysis and whole-genome sequencing for Enterohemorrhagic *Escherichia coli* O157. Appl Environ Microbiol.

[31] Ford L, Carter GP, Wang Q, Seemann T, Sintchenko V (2018). Incorporating whole-genome sequencing into public health surveillance: lessons from prospective sequencing of *Salmonella* Typhimurium in Australia. Foodborne Pathog Dis.

[32] Kwong JC, McCallum N, Sintchenko V, Howden BP (2015). Whole genome sequencing in clinical and public health microbiology. Pathology.

[33] Köser CU, Ellington MJ, Cartwright EJP, Gillespie SH, Brown NM (2012). Routine use of microbial whole genome sequencing in diagnostic and public health microbiology. PLoS Pathog.

[34] Lieberman TD, Flett KB, Yelin I, Martin TR, McAdam AJ (2014). Genetic variation of a bacterial pathogen within individuals with cystic fibrosis provides a record of selective pressures. Nat Genet.

[35] Djeghout B, Bloomfield SJ, Rudder S, Elumogo N, Mather AE (2022). Comparative genomics of *Campylobacter jejuni* from clinical campylobacteriosis stool specimens. Gut Pathog.

[36] Eyre DW, Cule ML, Griffiths D, Crook DW, Peto TEA (2013). Detection of mixed infection from bacterial whole genome sequence data allows assessment of its role in *Clostridium difficile* transmission. PLoS Comput Biol.

[37] Wilkinson DJ, Dickins B, Robinson K, Winter JA (2022). Genomic diversity of populations from different regions of the human stomach. Gut Microbes.

[38] Liu QY, Via LE, Luo T, Liang LL, Liu X (2015). Within patient microevolution of correlates with heterogeneous responses to treatment. Sci Rep-Uk.

[39] Raghuram V, Gunoskey JJ, Hofstetter KS, Jacko NF, Shumaker MJ (2023). Comparison of genomic diversity between single and pooled *Staphylococcus aureus* colonies isolated from human colonization cultures. Microb Genom.

[40] Paterson GK, Harrison EM, Murray GGR, Welch JJ, Warland JH (2015). Capturing the cloud of diversity reveals complexity and heterogeneity of MRSA carriage, infection and transmission. Nat Commun.

[41] Tonkin-Hill G, Ling C, Chaguza C, Salter SJ, Hinfonthong P (2022). Pneumococcal within-host diversity during colonization, transmission and treatment. Nat Microbiol.

[42] Trees E, Carleton HA, Folster JP, Gieraltowski L, Hise K (2024). Genetic diversity in *Salmonella enterica* in outbreaks of foodborne and zoonotic origin in the USA in 2006–2017. Microorganisms.

[43] Prevention ECfD, Control EFSA (2023). Three clusters of *Salmonella* Enteritidis ST11 infections linked to chicken meat and chicken meat products. EFS3.

[44] Ghaderi R, Tadayon K, Khaki P, Mosavari N (2015). Iranian clonal population of *Salmonella enterica* serovar Enteritidis, characterized by multi-locus sequence typing (MLST) method. Iran J Microbiol.

[45] Lei C-W, Zhang Y, Kang Z-Z, Kong L-H, Tang Y-Z (2020). Vertical transmission of *Salmonella* Enteritidis with heterogeneous antimicrobial resistance from breeding chickens to commercial chickens in China. Vet Microbiol.

[46] Castellanos LR, van der Graaf-van Bloois L, Donado-Godoy P, Veldman K, Duarte F (2020). Antimicrobial resistance in *Salmonella enterica* Serovar Paratyphi B Variant Java in Poultry from Europe and Latin America. *Emerg Infect Dis*.

[47] Chung The H, Pham P, Ha Thanh T, Phuong LVK, Yen NP (2023). Multidrug resistance plasmids underlie clonal expansions and international spread of *Salmonella enterica* serotype 1,4,[5],12:i:- ST34 in Southeast Asia. *Commun Biol*.

[48] Branchu P, Bawn M, Kingsley RA (2018). Genome variation and molecular epidemiology of *Salmonella enterica* serovar typhimurium pathovariants. Infect Immun.

[49] Desai PT, Porwollik S, Long F, Cheng P, Wollam A (2013). Evolutionary genomics of *Salmonella enterica* subspecies. mBio.

[50] Page AJ, Ainsworth EV, Langridge GC (2020). *socru*: typing of genome-level order and orientation around ribosomal operons in bacteria. Microb Genom.

[51] Waters EV, Tucker LA, Ahmed JK, Wain J, Langridge GC (2022). Impact of *Salmonella* genome rearrangement on gene expression. *Evol Lett*.

[52] Waters EV, Lee WWY, Ismail Ahmed A, Chattaway MA, Langridge GC (2024). From acute to persistent infection: revealing phylogenomic variations in *Salmonella* Agona. PLoS Pathog.

[53] Ewels P, Magnusson M, Lundin S, Käller M (2016). MultiQC: summarize analysis results for multiple tools and samples in a single report. Bioinformatics.

[54] Chen SF, Zhou YQ, Chen YR, Gu J (2018). fastp: an ultra-fast all-in-one FASTQ preprocessor. Bioinformatics.

[55] Filtlong WR (2017). GitHub repository.

[56] Wick RR, Judd LM, Gorrie CL, Holt KE (2017). Unicycler: resolving bacterial genome assemblies from short and long sequencing reads. PLoS Comput Biol.

[57] Tatusova T, DiCuccio M, Badretdin A, Chetvernin V, Nawrocki EP (2016). NCBI prokaryotic genome annotation pipeline. Nucleic Acids Res.

[58] Giardine B, Riemer C, Hardison RC, Burhans R, Elnitski L (2005). Galaxy: a platform for interactive large-scale genome analysis. Genome Res.

[59] Zhang S, den Bakker HC, Li S, Chen J, Dinsmore BA (2019). SeqSero2: rapid and improved *Salmonella* serotype determination using whole-genome sequencing data. Appl Environ Microbiol.

[60] Yoshida CE, Kruczkiewicz P, Laing CR, Lingohr EJ, Gannon VPJ (2016). The *Salmonella* In Silico Typing Resource (SISTR): an open web-accessible tool for rapidly typing and subtyping draft *Salmonella* genome assemblies. PLoS One.

[61] Carver T, Berriman M, Tivey A, Patel C, Böhme U (2008). Artemis and ACT: viewing, annotating and comparing sequences stored in a relational database. Bioinformatics.

[62] Nguyen L-T, Schmidt HA, von Haeseler A, Minh BQ (2015). IQ-TREE: a fast and effective stochastic algorithm for estimating maximum-likelihood phylogenies. Mol Biol Evol.

[63] Minh BQ, Schmidt HA, Chernomor O, Schrempf D, Woodhams MD (2020). IQ-TREE 2: new models and efficient methods for phylogenetic inference in the genomic era. Mol Biol Evol.

[64] Parks DH, Imelfort M, Skennerton CT, Hugenholtz P, Tyson GW (2015). CheckM: assessing the quality of microbial genomes recovered from isolates, single cells, and metagenomes. Genome Res.

[65] Zhou ZM, Alikhan NF, Mohamed K, Fan YL, Achtman M (2020). The enterobase user’s guide, with case studies on transmissions, phylogeny, and core genomic diversity. Genome Res.

[66] Song S, Hwang S, Lee S, Ha NC, Lee K (2014). Interaction mediated by the putative tip regions of MdsA and MdsC in the formation of a *Salmonella*-specific tripartite efflux pump. PLoS One.

[67] Czibener C, Merwaiss F, Guaimas F, Del Giudice MG, Serantes DAR (2016). BigA is a novel adhesin of Brucella that mediates adhesion to epithelial cells. Cell Microbiol.

[68] Berdejo D, Mortier J, Cambré A, Sobota M, Van Eyken R (2024). Evolutionary trade-off between heat shock resistance, growth at high temperature, and virulence expression in *Salmonella* Typhimurium. mBio.

[69] Gicquelais RE, Morris JF, Matthews HS, Gladden L, Safi H (2014). Multiple-serotype salmonella outbreaks in two state prisons—Arkansas, August 2012. MMWR Morb Mortal Wkly Rep.

[70] Mank L, Mandour M, Rabatsky-Ehr T, Phan Q, Krasnitski J (2010). Multiple-serotype *Salmonella* gastroenteritis outbreak after a reception--Connecticut, 2009. MMWR Morb Mortal Wkly Rep.

[71] Matthews TD, Rabsch W, Maloy S (2011). Chromosomal rearrangements in *Salmonella enterica* serovar Typhi strains isolated from asymptomatic human carriers. mBio.

[72] Waldram A, Dolan G, Ashton PM, Jenkins C, Dallman TJ (2018). Epidemiological analysis of Salmonella clusters identified by whole genome sequencing, England and Wales 2014. Food Microbiol.

[73] Miller KA, Phillips RS, Mrázek J, Hoover TR (2013). Salmonella utilizes D-glucosaminate via a mannose family phosphotransferase system permease and associated enzymes. J Bacteriol.

[74] Erni B, Zanolari B, Kocher HP (1987). The mannose permease of *Escherichia coli* consists of three different proteins. Amino acid sequence and function in sugar transport, sugar phosphorylation, and penetration of phage lambda DNA. J Biol Chem.

[75] Erni B, Zanolari B, Graff P, Kocher HP (1989). Mannose permease of *Escherichia-coli* - domain-structure and function of the phosphorylating subunit. Biol Chem H-S.

[76] Pajand O, Rahimi H, Darabi N, Roudi S, Ghassemi K (2021). Arrangements of mobile genetic elements among virotype E subpopulation of *Escherichia coli* sequence type 131 strains with high antimicrobial resistance and virulence gene content. mSphere.

[77] Sekizuka T, Lee K, Kimata K, Isobe J, Kuroda M (2019). Complete genome sequence of an Enterohemorrhagic *Escherichia coli* O111:H8 strain recovered from a large outbreak in Japan associated with consumption of raw beef. *Microbiol Resour Announc*.

[78] Nair S, Chattaway M, Langridge GC, Gentle A, Day M (2021). ESBL-producing strains isolated from imported cases of enteric fever in England and Wales reveal multiple chromosomal integrations of blaCTX-M-15 in XDR *Salmonella* Typhi. J Antimicrob Chemother.

[79] Mäklin T, Kallonen T, Alanko J, Samuelsen Ø, Hegstad K (2021). Bacterial genomic epidemiology with mixed samples. Microb Genom.

[80] Holt KE, Teo YY, Li H, Nair S, Dougan G (2009). Detecting SNPs and estimating allele frequencies in clonal bacterial populations by sequencing pooled DNA. Bioinformatics.

[81] Gilchrist CLM, Chooi Y-H (2021). Clinker & clustermap.js: automatic generation of gene cluster comparison figures. Bioinformatics.

