# Supplementary material for: Genomic diversity of non-typhoidal Salmonella found in patients suffering from gastroenteritis in Norfolk, UK
Source: Microb Genom. 2025 Aug 11;11(8):001468. doi: 10.1099/mgen.0.001468 (PMC12452188; doi:10.1099/mgen.0.001468)
Supplement: Supplementary Material 2. [file mgen-11-01468-s002.pdf]

Table S1. Patient demographics and sample collection details for *Salmonella*-positive cases in Norwich, UK, 2020-2022

Patient ID	EPA result	Date sample collected	Date QIB Collected	Sample origin (GP vs. Outpatient)	Age	Sex	Recent travel	Travel-region
1	Sal+	13/01/2020	14/01/2020	GP	31	F	Yes	Thailand
2	Sal+	03/03/2020	03/03/2020	GP	62	F	No	
3	Sal+	21/08/2020	24/08/2020	GP	65	M	No	
4	Sal+	18/02/2022	21/02/2022	Outpatient	2	F	No	
5	Sal+	20/05/2022	23/05/2022	GP	77	F	No	
6	Sal+	25/06/2022	27/06/2022	Outpatient	29	F	Yes	South Africa
7	Sal+	20/07/2022	25/07/2022	GP	44	F	No	
8	Sal+	08/08/2022	10/08/2022	Outpatient	55	F	na	

Sal+ = Salmonella positive, **GP =** General practitioner**, EPA=** Eastern Pathology Alliance, **QIB =** Quadram Institute Bioscience**, M =** Male, **F** = Female, **na** = Not available

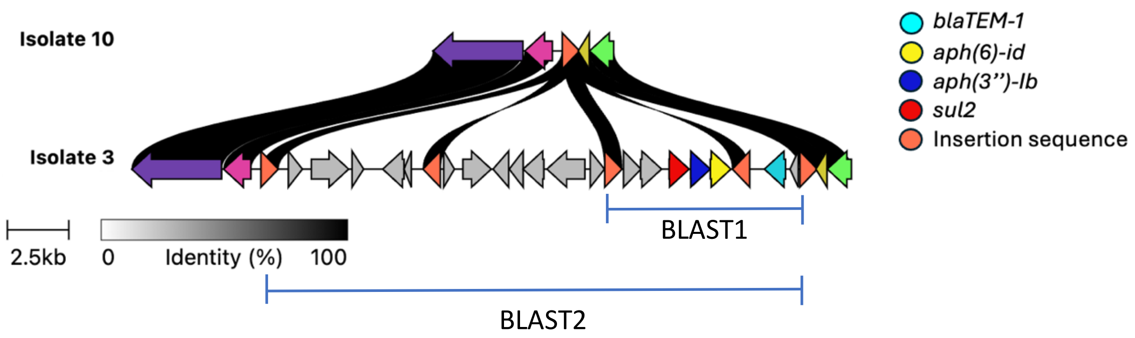

BLAST search 1 (Query Length 8647bp)

Escherichia coli O111:H- 110512 plasmid pO111-110512_1 DNA, complete genome, AP019762.1. Score 15969 bits(8647), Identities 8647/8647(100%), Gaps 0/8647(0%).

atgaacccattcaaa

ggccggcattttcagcgtgacatcattctgtgggccgtacgctggtactgcaaatacggc

atcagttaccgtgagctgcaggagatgctggctgaacgcggagtgaatgtcgatcactcc

acgatttaccgctgggttcagcgttatgcgcctgaaatggaaaaacggctgcgctggtac

tggcgtaacccttccgatctttgcccgtggcacatggatgaaacctacgtgaaggtcaat

ggccgctgggcgtatctgtaccgggccgtcgacagccggggccgcactgtcgatttttat

ctctcctcccgtcgtaacagcaaagctgcataccggtttctgggtaaaatcctcaacaac

gtgaagaagtggcagatcccgcgattcatcaacacggataaagcgcccgcctatggtcgc

gcgcttgctctgctcaaacgcgaaggccggtgcccgtctgacgttgaacaccgacagatt

aagtaccggaacaacgtgattgaatgcgatcatggcaaactgaaacggataatcggcgcc

acgctgggatttaaatccatgaagacggcttacgccaccatcaaaggtattgaggtgatg

cgtgcactacgcaaaggccaggcctcagcattttattatggtgatcccctgggcgaaatg

cgcctggtaagcagagtttttgaaatgtaaggcctttgaataagacaaaaggctgcctca

tcgctaactttgcaacagtgccggtaaatccatgctggccctgcaactggccgcacagat

tgcaggcgggccggatctgctggaggtgggcgaactgcccaccggcccggtgatctacct

gcccgccgaagacccgcccaccgccattcatcaccgcctgcacgcccttggggcgcacct

cagcgccgaggaacggcaagccgtggctgacggcctgctgatccagccgctgatcggcag

cctgcccaacatcatggccccggagtggttcgacggcctcaagcgcgccgccgagggccg

ccgcctgatggtgctggacacgctgcgccggttccacatcgaggaagaaaacgccagcgg

ccccatggcccaggtcatcggtcgcatggaggccatcgccgccgataccgggtgctctat

cgtgttcctgcaccatgccagcaagggcgcggccatgatgggcgcaggcgaccagcagca

ggccagccggggcagctcggtactggtcgataacatccgctggcagtcctacctgtcgag

catgaccagcgccgaggccgaggaatggggtgtggacgacgaccagcgccggttcttcgt

ccgcttcggtgtgagcaaggccaactatggcgcaccgttcgctgatcggtggttcaggcg

gcatgacggcggggtgctcaagcccgccgtgctggagaggcagcgcaagagcaagggggt

gccccgtggtgaagcctaagaacaagcacagcctcagccacgtccggcacgacccggcgc

actgtctggcccccggcctgttccgtgccctcaagcggggcgagcgcaagcgcagcaagc

tggacgtgacgtatgactacggcgacggcaagcggatcgagttcagcggcccggagccgc

tgggcgctgatgatctgcgcatcctgcaagggctggtggccatggctgggcctaatggcc

tagtgcttggcccggaacccaagaccgaaggcggacggcagctccggctgttcctggaac

ccaagtgggaggccgtcaccgctgatgccatggtggtcaaaggtagctatcgggcgctgg

caaaggaaatcggggcagaggtcgatagtggtggggcgctcaagcacatacaggactgca

tcgagcgcctttggaaggtatccatcatcgcccagaatggccgcaagcggcaggggtttc

ggctgctgtcggagtacgccagcgacgaggcggacgggcgcctgtacgtggccctgaacc

ccttgatcgcgcaggccgtcatgggtggcggccagcatgtgcgcatcagcatggacgagg

tgcgggcgctggacagcgaaaccgcccgcctgctgcaccagcggctgtgtggctggatcg

accccggcaaaaccggcaaggcttccatagataccttgtgcggctatgtctggccgtcag

aggccagtggttcgaccatgcgcaagcgccgccagcgggtgcgcgaggcgttgccggagc

tggtcgcgctgggctggacggtaaccgagttcgcggcgggcaagtacgacatcacccggc

ccaaggcggcaggctgacccccccccactctattgtaaacaagacatttttatcttttata

ttcaatggcttattttcctgctaattggtaataccatgaaaaataccatgctcagaaaag

gcttaacaatattttgaaaaattgcctactgagcgctgccgcacagctccataggccgct

ttcctggctttgcttccagatgtatgctattctgctcctgcagctaatggatcaccgcaa

acaggttactcgcctggggattccctttcgacccgagcatccgtatgagactcatgctcg

attattattattatagaagcccccatgaataaatcgctcatcattttcggcatcgtcaac

ataacctcggacagtttctccgatggaggccggtatctggcgccagacgcagccattgcg

caggcgcgtaagctgatggccgagggggcagatgtgatcgacctcggtccggcatccagc

aaccccgacgccgcgcctgtttcgtccgacacagaaatcgagcgtatcgcgccggtgctg

gacgcgctcaaggcagatggcattcccgtctcgctcgacagttatcaacccgcgacgcaa

gcctatgccttgtcgcgtggtgtggcctatctcaatgatattcgcggttttccagacgct

gcgttctatccgcaattggcgaaatcatctgccaaactcgtcgttatgcattcggtgcaa

gacgggcaggcagatcggcgcgaggcacccgctggcgacatcatggatcacattgcggcg

ttctttgacgcgcgcatcgcggcgctgacgggtgccggtatcaaacgcaaccgccttgtc

cttgatcccggcatggggtttttctgggggctgctcccgaaacctcgctctcggtgctg

gcgcggttcgatgaattgcggctgcgcttcgatttgccggtgcttctgtctgtttcgcgc

aaatcctttctgcgcgcgctcacaggccgtggtccgggggatgtcggggccgcgacactc

gctgcagagcttgccgccgccgcaggtggagctgacttcatccgcacacacgagccgcgc

cccttgcgcgacgggctggcggtattggcggcgctaaaagaaaccgcaagaattcgttaa

ctgcacattcgggatatttctctatattcgcgcttcatcagaaaactgaaggaacctcca

ttgaatcgaactaatattttttttggtgaatcgcattctgactggttgcctgtcagaggc

ggagaatctggtgattttgtttttcgacgtggtgacgggcatgccttcgcgaaaatcgca

cctgcttcccgccgcggtgagctcgctggagagcgtgaccgcctcatttggctcaaaggt

cgaggtgtggcttgccccgaggtcatcaactggcaggaggaacaggagggtgcatgcttg

gtgataacggcaattccgggagtaccggcggctgatctgtctggagcggatttgctcaaa

gcgtggccgtcaatggggcagcaacttggcgctgttcacagcctatcggttgatcaatgt

ccgtttgagcgcaggctgtcgcgaatgttcggacgcgccgttgatgtggtgtcccgcaat

gccgtcaatcccgacttcttaccggacgaggacaagagtacgccgcagctcgatcttttg

gctcgtgtcgaacgagagctaccggtgcggctcgaccaagagcgcaccgatatggttgtt

tgccatggtgatccctgcatgccgaacttcatggtggaccctaaaactcttcaatgcacg

ggtctgatcgaccttgggcggctcggaacagcagatcgctatgccgatttggcactcatg

attgctaacgccgaagagaactgggcagcgccagatgaagcagagcgcgccttcgctgtc

ctattcaatgtattggggatcgaagcccccgaccgcgaacgccttgccttctatctgcga

ttggaccctctgacttggggttgatgttcatgccgcctgtttttcctgctcattggcacg

tttcgcaacctgttctcattgcggacaccttttccagcctcgtttggaaagtttcattgc

cagacgggactcctgcaatcgtcaagggattgaaacctatagaagacattgctgatgaac

tgcgcggggccgactatctggtatggcgcaatgggaggggagcagtccggttgctcggtc

gtgagaacaatctgatgttgctcgaatatgccggggagcgaatgctctctcacatcgttg

ccgagcacggcgactaccaggcgaccgaaattgcagcggaactaatggcgaagctgtatg

ccgcatctgaggaacccctgccttctgcccttctcccgatccgggatcgctttgcagctt

tgtttcagcgggcgcgcgatgatcaaaacgcaggttgtcaaactgactacgtccacgcgg

cgattatagccgatcaaatgatgagcaatgcctcggaactgcgtgggctacatggcgatc

tgcatcatgaaaacatcatgttctccagtcgcggctggctggtgatagatcccgtcggtc

tggtcggtgaagtgggctttggcgccgccaatatgttctacgatccggctgacagagacg

acctttgtctcgatcctagacgcattgcacagatggcggacgcattctctcgtgcgctgg

acgtcgatccgcgtcgcctgctcgaccaggcgtacgcttatgggtgcctttccgcagctt

ggaacgcggatggagaagaggagcaacgcgatctagctatcgcggccgcgatcaagcagg

tgcgacagacgtcatactagatatcaagggcactgttgcaaagttagcgatgaggcagcc

ttttgtcttattcaaaggccttacatttcaaaaactctgcttaccaggcgcatttcgccc

aggggatcaccataataaaatgctgaggcctggcctttgcgtagtgcacgcatcacctca

atacctttgatggtggcgtaagccgtcttcatggatttaaatcccagcgtggcgccgatt

atccgtttcagtttgccatgatcgcattcaatcacgttgttccggtacttaatctgtcgg

tgttcaacgtcagacgggcaccggccttcgcgtttgagcagagcaagcgcgcgaccatag

gcgggcgctttatccgtgttgatgaatcgcgggatctgccacttcttcacgttgttgagg

attttacccagaaaccggtatgcagctttgctgttacgacgggaggagagataaaaatcg

acagtgcggccccggctgtcgacggcccggtacagatacgcccagcggccattgaccttc

acgtaggtttcatccatgtgccacgggcaaagatcggaagggttacgccagtaccagcgc

agccgtttttccatttcaggcgcataacgctgaacccagcggtaaatcgtggagtgatcg

acattcactccgcgttcagccagcatctcctgcagctcacggtaactgatgccgtatttg

cagtaccagcgtacggcccacagaatgatgtcacgctgaaaatgccggcctttgaatggg

ttcatgtgcagctccatcagcaaaaggggatgataagtttatcaccaccgactatttgca

acagtgccatatcaagcgacttctcctatcccctgggaacacatcaatctcaccggagaa

tatcgctggccaaagccttagcgtaggattccgccccttcccgcaaacgaccccaaacag

gaaacgcagctgaaacgggaagctcaacacccactgacgcatgggttgttcaggcagtac

ttcatcaaccagcaaggcggcactttcggccatccgccgcgccccacagctcgggcagaa

accgcgacgcttacagctgaaagcgaccaggtgctcggcgtggcaagactcgcagcgaac

ccgtagaaagccatgctccagccgcccgcattggagaaattcttcaaattcccgttgcac

atagcccggcaattcctttccctgctctgccataaggggtctgacgctcagtggaacgaa

aactcacgttaagggattttggtcatgagattatcaaaaaggatcttcacctagatcctt

ttaaattaaaaatgaagttttaaatcaatctaaagtatatatgagtaaacttggtctgac

agttaccaatgcttaatcagtgaggcacctatctcagcgatctgtctatttcgttcatcc

atagttgcctgactccccgtcgtgtagataactacgatacgggagggcttaccatctggc

cccagtgctgcaatgataccgcgagacccacgctcaccggctccagatttatcagcaata

aaccagccagccggaagggccgagcgcagaagtggtcctgcaactttatccgcctccatc

cagtctattaattgttgccgggaagctagagtaagtagttcgccagttaatagtttgcgc

aacgttgttgccattgctgcaggcatcgtggtgtcacgctcgtcgtttggtatggcttca

ttcagctccggttcccaacgatcaaggcgagttacatgatcccccatgttgtgcaaaaaa

gcggttagctccttcggtcctccgatcgttgtcagaagtaagttggcagcagtgttatca

ctcatggttatggcagcactgcataattctcttactgtcatgccatccgtaagatgcttt

tctgtgactggtgagtactcaaccaagtcattctgagaatagtgtatgcggcgaccgagt

tgctcttgcccggcgtcaacacgggataataccgcaccacatagcagaactttaaaagtg

ctcatcattggaaaacgttcttcggggcgaaaactctcaaggatcttaccgctgttgaga

tccagttcgatgtaacccactcgtgcacccaactgatcttcagcatcttttactttcacc

agcgtttctgggtgagcaaaaacaggaaggcaaaatgccgcaaaaaagggaataagggcg

acacgaaaatgttgaatactcatactcttcctttttcaatattattgaagcatttaccag

ggttattgtctcatgagcggatacatatttgaatgtatttagaaaaataaacaaataggg

gttccgcgcacatttccccgaaaagtgccacctgacgtctaagaaaccattattatcatg

acattaacctataaaaataggcgtatcacgaggccctttcgtcttcaagaattttataaa

ccgtggagcgggcaatactgagctgatgagcaatttccgttgcaccagtgcccttctgat

gaagcgtcagcacgacgttcctgtccacggtacgcctgcggccaaatttgattcctttca

gctttgcttcctgtcggccctcattcgtgcgttctaggatcctccggcgttcagcctgtg

ccacagccgacaggatggtgaccaccatttgccccatatcaccgtcggtactgatcccgt

catcaatgaaccggactgccacgccctgagcgtcaaattcctttatcagttggatcatat

cggcggcactgttgcaaatagtcggtggtgataaacttatcatccccttttgctgatgga

gctgcacatgaacccattcaaaggccggcattttcagcgtgacatcattctgtgggccgt

acgctggtactgcaaatacggcatcagttaccgtgagctgcaggagatgctggctgaacg

cggagtgaatgtcgatcactccacgatttaccgctgggttcagcgttatgcgcctgaaat

ggaaaaacggctgcgctggtactggcgtaacccttccgatctttgcccgtggcacatgga

tgaaacctacgtgaaggtcaatggccgctgggcgtatctgtaccgggccgtcgacagccg

gggccgcactgtcgatttttatctctcctcccgtcgtaacagcaaagctgcataccggtt

tctgggtaaaatcctcaacaacgtgaagaagtggcagatcccgcgattcatcaacacgga

taaagcgcccgcctatggtcgcgcgcttgctctgctcaaacgcgaaggccggtgcccgtc

tgacgttgaacaccgacagattaagtaccggaacaacgtgattgaatgcgatcatggcaa

actgaaacggataatcggcgccacgctgggatttaaatccatgaagacggcttacgccac

catcaaaggtattgaggtgatgcgtgcactacgcaaaggccaggcctcagcattttatta

tggtgatccccctgggcgaaatgcgcctggtaagcagagtttttgaaatgtaa

BLAST search 2 (Query Length 22636bp)

Escherichia coli O111:H- 110512 plasmid pO111-110512_1 DNA, complete genome, AP019762.1. Score 16085 bits(8710), Identities 8710/8710(100%), Gaps 0/8710(0%).

atgaacccattcaaaggccggcattttcagcgtgac

atcattctgtgggccgtacgctggtactgcaaatacggcatcagttaccgtgagctgcag

gagatgctggctgaacgcggagtgaatgtcgatcactccacgatttaccgctgggttcag

cgttatgcgcctgaaatggaaaaacggctgcgctggtactggcgtaacccttccgatctt

tgcccgtggcacatggatgaaacctacgtgaaggtcaatggccgctgggcgtatctgtac

cgggccgtcgacagccggggccgcactgtcgatttttatctctcctcccgtcgtaacagc

aaagctgcataccggtttctgggtaaaatcctcaacaacgtgaagaagtggcagatcccg
cgattcatcaacacggataaagcgcccgcctatggtcgcgcgcttgctctgctcaaacgc
gaaggccggtgcccgtctgacgttgaacaccgacagattaagtaccggaacaacgtgatt
gaatgcgatcatggcaaactgaaacggataatcggcgccacgctgggatttaaatccatg
aagacggcttacgccaccatcaaaggtattgaggtgatgcgtgcactacgcaaaggccag
gcctcagcattttattatggtgatcccctgggcgaaatgcgcctggtaagcagagttttt
gaaatgtaaggcctttgaataagacaaaaggctgcctcatcgctaactttgcaacagtgc
cgtattgataacacgttgcggggcaatttaatggtgtccggcggcagatcgtgttctgtg
cagactttccggcgttaaatcccgtatcacctgcgagcctggactatctctgtggtatca
actctgtaggaattttgtccccttcgtcttcattagccaatgaaaccggctttaacgtca
ccgttcgaatgttgcgaacgaacagctccttatgcggaatatcgtcaccgctctgcgatt
tttatagcgcatcagccacacgatttattggttcttgaaaaccaaggtttttgataaagc
aatcctccatgagaaaagcgactaaaattcttccttatctgatgtaaaggagaaaatcat
ggctactattgggtatattcgggtgtcaacaattgaccaaaatatcgatttacagcgtaa
tgcgcttactagtgcaaattgtgaccgcatttttgaggaccgtatcagtggcaagattgc
aaaccgccccggcctgaaacgagcgttaaagtatgtaaataaaggcgatactcttgtcgt
ctggaaattagacagactgggccgcagcgtgaaaaacctggtggcgttaatatcagaatt
acatgaacgtggagctcacttccattctttaaccgatagtattgataccagtagcgcgat
ggggcgattctttttcatgtaatgtcagcactggccgagatggagcgagaattaattgt
cgagcgaacccttgccggactggctgccgccagagcgcaaggacgactgggagggcgccc
tcgggcgatcaacaaacatgaacaggaacagattagtcggctattagagaaaggccatcc
tcggcagcaactagctattatttttggtattggcgtatctaccttatacagatattttcc
ggcaagccgcataaaaaaacgaatgaattaaaataaaaattacaacaggatggatataac
atttttgtaatacaggcgtgtggcataaataaaccgaaagggtatacaaaaaagacagca
tctaattaaaaagagaaaaaattcaacgtattaacatatatagtgtaacgcgctcacgat
aaggcctatgttacatccagctatagacgacatcgctcaaaacactaccagacacagtat
tcacctggaaaggctttttaatcaaaatgttagatgtaagcaattacggacagaaaaaat
agtaaagtttatgcctcaagtgtcgataacctggatgacacaggtaagcctggcataaca
ttggttatcaaaaaccttccaaaaggaaaattttatggcacaagtaatcaacactaacag
tctgtcgctgctgacccagaataacctgaacaaatcccagtccgcactgggcaccgctat
cgagcgtctgtcttctggtctgcgtatcaacagcgcgaaagacgatgcggcaggtcaggc
gattgctaaccgtttcaccgcgaacatcaaaggtctgactcaggcttcccgtaacgctaa
cgacggtatctccattgcgcagaccactgaaggcgcgctgaacgaaatcaacaacaacct

gcagcgtgtgcgtgaactggcggttcagtctgctaacagcaccaactcccagtctgacct
cgactccatccaggctgaaatcacccagcgcctgaacgaaatcgaccgtgtatccggcca
gactcagttcaacggcgtgaaagtcctggcgcaggacaacaccctgaccatccaggttgg
cgccaacgacggtgaaactatcgatatcgatctgaagcagatcaactctcagaccctggg
tctggactcactgaacgtgcagaaagcgtatgatgtgaaagatacagcagtaacaacgaa
agcttatgccaataatggtactacactggatgtatcgggtcttgatgatgcagctattaa
agcggctacgggtggtacgaatggtacggcttctgtaaccggtggtgcggttaaatttga
cgcagataataacaagtactttgttactattggtggctttactggtgctgatgccgccaa
aaatggcgattatgaagttaacgttgctactgacggtacagtaacccttgcggctggcgc
aactaaaaccacaatgcctgctggtgcgacaactaaaacagaagtacaggagttaaaaga
tacaccggcagttgtttcagcagatgctaaaaatgccttaattgctggcggcgttgacgc
taccgatgctaatggcgctgagttggtcaaaatgtcttataccgataaaaatggtaagac
aattgaaggcggttatgcgcttaaagctggcgataagtattacgccgcagattacgatga
agcgacaggagcaattaaagctaaaaccacaagttatactgctgctgacggcactaccaa
aacagcggctaaccaactgggtggcgtagacggtaaaaccgaagtcgttactatcgacgg
taaaacctacaatgccagcaaagccgctggtcatgatttcaaagcacaaccagagctggc
ggaagcagccgctaaaaccaccgaaaacccgctgcagaaaattgatgccgcgctggcgca
ggtggatgcgctgcgctctgatctgggtgcggtacaaaaccgtttcaactctgctatcac
caacctgggcaataccgtaaacaatctgtctgaagcgcgtagccgtatcgaagattccga
ctacgcgaccgaagtttccaacatgtctcgcgcgcagattctgcagcaggccggtacttc
cgttctggcgcaggctaaccaggtcccgcagaacgtgctgtctctgttacgttaatttat
ttcgttttattcagccccgtgaattcggggctttttcatttagcatagatgaatatatat
ttatggaatgtatggctgtaaatgatatttcctacgggcgagaagctgaaatatggccgc
gggattattctatgcttgctcgtcgagttcaatttctacgttttaatgatatccctgttc
gattggtgagtaataatgcccggataatcacaggctacattgcgaagtttaatccgaagg
aaaatttgattctggcttcggataaacctaaaggaaataagcgcattgaagttaaactag
agtctctggcaattcttgaagaattatcaggtaatgacgcttttaatctttcgctggtgc
cggctgacggatttaatcttcagcaatatactccatcaagaagagattatttctcgattt
gcaataagtgctataaacagggagtcggtatcaaaatctatatgaagtatggacaggttt
tgactggcaaaacgacaggcgtaaatgcgtgtcaggttggtgtgaggacatccaatggca
atcatatgcaagttatgtttgactgggtgagcaggatcacgtcttcggactacgctgaat
aacgcctacggtaataaaaaattccgtgagaaaagtaaaacttagggggctaccggaggg
gacctaatgaacggaggtcatggaaggtattcatcgtgccagactcttgctcttgtcaga

agaaggtaaaagtaggttccgcgacgcatttgggttcacgggcatcataagagagatgaa

cactgataaagtactttctcctgggataccggaacagtccaggaaagagggcagttacac

tattgtcgtctggtgatgattactcttcattcatgcttgccgaacgagttgaagcgatgt

aagaggccgtagctatcgatcccaataatcccaaaatttaccaggccttttatgaaggct

caaatttggctcaatgggtacgccaacgccttctgtgatactgtaggggttccaagtttt

acatagtgtctaatttaatgctatttgtgggttgataacccaactcacttcgaactggtt

tgtcgatttgctgaagttctcaatttgcctgaaggttaattctacgcgctggatgacagc

tttgctgatgcaatgctgaagctatatgggggaaaacgggtgcagtggaaaaggtgcata

ataagttaattggtcgtgttagtacccccctttaatgttatggtttctatctttatgaaa

tctaaaaataaaagaacttgagcattactaaatattaccgtgaaatccctataaggtgca

cggtatgatccccaaaagactgaaggaagcttgatcctacccgcgtaatatgggcacaac

cctaagcgaggttctggttttcaaattgttccggactgagaccgccacaggcactgtgac

gacgccaccgattgtaatcacactcactataattaaacactgctgtccgcattatttccc

ggctgacaaagtcctctccgtggatacattccaccctcagcgtatggaagaagctttccg

cacaggcattgtcgtaacaacaacctctggcgctcatacttccccgcagattatggcgtt

tcagtaagctctggtaatccgttgaacagtactgaccgcctctgtctgtatgcacgatga

cattttccggacatttacgctgccacagcgccatctgtaacgcattgcaggcaagctgtg

ctgtcatccgcgaggacatcgaccagccaatgactgaccgcggccacagatcgataacca

cggccagataaagccagccttcaccagtgcgaagatacgtgatgtcacccacccacttct

gattcgggccgctggcgtaaaagtcctgcttcagcagattctctgagactggcagaccat

gtttgcggtaactgaccggacggaaccggcgcgaggctttcgcccgcagcccctgccgac

gcaggctggccgccacggttttcacgttgaactggtaaccctgagcacgaagctcatccg

tcaggcgtggcgcaccatagcgttgttttgcgtcactgaatgcttcccggacgacgttat

cacagacaaggcggaaccgctgacgccggtttatctgatgacgacgctgatgccagacgt

accagccgctacgggcaacctgaaatacacggcacatggctttgatattgaactcagcct

gatgtttttcgatgaagacatacttcatttcaggcgcttcgcgaagtatgtcgcggcctt

ctggagaatggccagttcctcatcccgttctgccagttgacgtttcagacgggcgatctc

agcggacatctcctgctcgcgttcagaagaagagagctgattttgctgtttgcttcgcca

gttgtggagctgtgatttatacaggctgagctcacgggctgcagcggcaacaccaatgcg

cttagccagtttcagggcttcctgacgaaattcaggcgtgtgctgcttgcgtggcttttt

ggtggttaatgctggttttgtcatgtgagtcacctcttacttgagagtttactcacttag

tcgcgtgtccactattgctgggtaagatcaggtaggtttaccctctcatcccaacgaata

acttacgatacgacattgcagagcaggctaaagtttatcggaacgcgagccaccttaacg

aagctgcaaagtgtttttttttctgtgtaaagagtatgattcagaacaaagacagtcatta
gctcgcacagcacacaactaagaatttgaaatgagtagacaacccgctaaagaggaagtt
taatgtccaactactgcttttactctcaggatgcattagctctggctcaaagtgctggtg
ttgatgtaataataaacagctatgctgagcagcataaaaaacaaacatatattctttgca
gacctctatctaatgaggatgtaaagggcactgttgcaaagttagcgatgaggcagcctt
ttgtcttattcaaaggccttacatttcaaaaactctgcttaccaggcgcatttcgcccag
gggatcaccataataaaatgctgaggcctggcctttgcgtagtgcacgcatcacctcaat
acctttgatggtggcgtaagccgtcttcatggatttaaatcccagcgtggcgccgattat
ccgtttcagtttgccatgatcgcattcaatcacgttgttccggtacttaatctgtcggtg
ttcaacgtcagacgggcaccggccttcgcgtttgagcagagcaagcgcgcgaccataggc
gggcgctttatccgtgttgatgaatcgcgggatctgccacttcttcacgttgttgaggat
tttacccagaaaccggtatgcagctttgctgttacgacgggaggagagataaaaatcgac
agtgcggccccggctgtcgacggcccggtacagatacgcccagcggccattgaccttcac
gtaggtttcatccatgtgccacgggcaaagatcggaagggttacgccagtaccagcgcag
ccgtttttccatttcaggcgcataacgctgaacccagcggtaaatcgtggagtgatcgac
attcactccgcgttcagccagcatctcctgcagctcacggtaactgatgccgtatttgca
gtaccagcgtacggcccacagaatgatgtcacgctgaaaatgccggcctttgaatgggtt
catgtgcagctccatcagcaaaaggggatgataagtttatcaccaccgactatttgcaac
agtgcccaagctccccggtgtggtttctcggttcccactgctttgacctggtgcgccacc
tctccgggcaggaggtggtatcggtgtacgccgtcggacagaagcggctgatggtcgaat
gcggcctcgacaccttcgacagcgtgcagagcctgctgacaatggccgacggcagttcgt
ggggggtggaaaactcatgggtgctgccggagggctttccgaaggacaacgacgggcgca
tcgacattctctgcgaggcaacctatatccgcagtacttcccaacatcgcgggctggaaa
tcaccacgccggccttgacgctcacgcccaacagctatttcatcaactaccgcaacggcg
ttgccagcggctttggtatcgacccgatcaatgattttgtgcgggcggtcagacataaag
ccccctacccggtcaccgccgaagacgggctggcggtgagcaggatctgcgaggcggtac
accgcagccttgagagcgggaaacctgaagatgtttaaacaaacggcgcagggtagctcc
ctgtgccagttccatatgattttgctaattctacttactggtctattcactcgtcaggct
ttggcccgcagatatattaatgttttccatcctgccgcaacaaaaccccacacacgattg
tacgtttttttagtcattaaggtgaatagcggtattttatggctatgaccttttttcacctt
cctgccaatagtatcatgctaaacctctcatttatgtttattgcgccaataatagtcaac
aagtatttcaaaaaaagtcccaagcaaattataatgctggagtttcataatgtccctttaa
aaaccattttaagaagagctattatgtccctccctacaaattttaatgatattcttcgtt

tttttgaaaaagattacgatacagccaaagaagataatgctttaagtgcacgcggccaat

ttctgcaactttaccccttaatcacctaaaaaaaatgacgcttgatgactatgtcatcg

gcaaaggtacagcttcattttgtgcttgtgttgaagtaaaaaccagaacatgggcaaata

tgcaaggtgcgacggcgctcaaatttggtatttattatggaaaatcaaaatcagatccaa

ccgtccgctatcgttttactcagaaatttggcgatgatgatagtactaataaagaagttt

tcgctaatgttaaagacgctttactagacctaatacagtcagggaaagaattagatttta

gagcgattgacgagaacccctatcccaaatgtttaaggctaaaatattgagcctttact

ttccagaacactttataaacatttgcagcaaagatcatcttaaagaaattgctatggaaa

tgggtataaaagagcaacagtttattagtaaatatcaacatttgttattcaagaaaaaac

tagagcataaaatcacccgaaactggagcaatccaaaatatatgtccttcctttatgccc

agttcatacgtaaggatcttagcagcgctcctgctgtgatagttaaaaaaccacaaaaaa

gaaaccatcccgaagtcaatttcgaagaaataacggacaatcgtgatttaataggcaaaa

aaagcgaagaatatgcattaaactgggaaaaaaaccgcctaatcggtctcggctattcaa

aactagctgaggaaatagatgatcgccgtaatcgtccaacttatggttacgactttcttt

cttttaatgccccaggtgatgagcgatacatcgaagttaaatcaattggccgggatggaa

aagagggagcattccgtttttcctctcaggaaatgaactcaccgtttctaatttaagta

accacagtaaaaactattatttttatcttgtacagtatgggaaagatggagagccatgca

atctatatgtaaaacatgctcaagatctttacactaatagtgaaatgtccccttgtgctt

acgttgttaggttcgatctggaagaacctgcttaatagtttcgacaacctccggctcata

actttaaaagtgtgaaatcaggcgcagagttccccctgcgccttcacctcagtgatgctg

taacaacacctgtgaaatcttcctcgcttcacctgccggatccgccgcatgcgtgatcgc

tgatcccacaatcactacatccgggcctaacagtgcgtagtccttcacagtctggctact

gataccgcctgccacggcaatccgggctttccggcgcaccttcaacatcgttatcaaatc

atcaatcggtttgcgccctgctgcctgctggtcggtgcctgtgtgtaccgccagcatatc

tgcccccgcctcctccagcaggcggactcgtgcggggagatcgtcgacgcagatcatatc

caccaccacctgctttccggcctcttttgccgcgcggatgcacgactggatcgtcagcac

gtcggtcacacctaacaccgtgacatagtctgccccggcgtcgaaaagcagctgcgattc

aaaatggccgccatccataattttcgcatccgccagtacttccttatgcgggtatttttc

tttaatggctttaatcgcgttcacgccttcccgaataagaaagggtgttcccacttcaat

aatatcaacgtcatcaaccaccttatccataaataccatcgcttcaggcagggttaactc

gtccagggcaagctgtaatttcatgttggtttccttgtgtcgaatgactattcgaggttg

gcgtgcagcgcaaagcctttggttaagcgatatcccgtcgactgcgccagattcacaatc

atggcgtcacccagcaccatgaccgcctcttcaaacaggctgccgccgggcagaatacct

ttcacattctcgggtccatccggcagtttgtcggaataagccgggatcctgaccgccacc
cccgccagtttccccagcgtggactcggggaaaatggtcagcaaggccacagtgtcaccc
agttgcttcgctttcgttgccacattcaccagcgaagccgtttcacccgatgcactcgcc
agtaacagcaggtcgcctttttgcagcgcaggcgtgaccacatcgccgacaacatgcacc
ttcaggccgatatgcatcaggcgcatcgcaaaagctttcagcatcaacagcgaacgccct
gcgccaaaaacaaataccgctttcgcgtcagcaatggcctgctccagacgcgccagcgca
gcgccatcaatccgcgacatcgcctgattcaggtcgctacaggcaacgcctgcaacagat
tgcgtttccatcatgcgtcctcctgacagagcgggaataaatcatcctcatactggagca
tccccatgatttcccggacatagttatcgacatgccccagaaaatgaaaacgttccggtg
cggcgcggtagcgctggtaaagcggcatatcgaaggtgatgccaagatgcgggttctctt
tgacggtggcgaatttgatgcgcggaacacgcgggtagtagtgattcttgcgcacgcaga
gctgcccgttctcctccacgatgtagcaggccataccgtggtaaaactgcaccgtgtcgt
agtgaaccgtacagggtttatacgccagattgctgaacaccagcggcccgtcgccgatat
tgatggagcagtggccataattcggcggcacgataatgctctgcccctccttcacgacgg
ccacgataagatcgtctaccaccagctcctgcggcgcggcggcaaaatccggcgactttt
gcaggatatacgccgccgtacccttaattacttcatacacctccggatgggtgttgcgcc
gcgtgtcgttatagccgtgataatggccactggttttcttacgctccttgccaatcgtcc
ccggcatgatgatggtgatgtcgtactgatactggtcagcagcaagccgttcctgatcct
ccgcaaagctgagaccgcgataaacatcgtaagccggttcattacgcagcccttcggtga
actccggtaaaaccaccgccatctgcccggcgcttttgcggccaaagccgagatagttta
gcggcggtttcagcgccattacgccatcatcatcgagatacagcggcaggccgctgtggt
gtagctgtttcatttttgccccctgtatacacgtcatacttcaagttgctgctacgttgg
ctgcgcgccttcatcccggtcacatagttatctatgctcccgggaactcacgcgcttgcc
gccttgctacaactcgaattatttagtgtatgggttcacaatgtccggaaaccggatgcg
ctttaatcgcgcggatcgcctctttcagccggtcggatttcatgccaaagacgctctgaa
tgttgttcccgacctcaacgacgcccgccgcgccaagctgtttcagcagcgctttgtcca
ccagcgtcttatccgtcacgtcaatgcgcagacgtgacatgcaggcttcgatgctggtca
tattccccaatccgccataggccagaatgatgccgctcaccaggtcgttctgttcgcttt
cctgcgccacagcggtttcttcgacttcacggcccggcgtcagcaaattccagcgacgga
tggcaaaggtaaacaggacgtagtacaccgcgcccatcaccagcccgaccgggaatacca
tgtaccagtgaggcgctcgcggcagaacgccaaagaagagatagtcgataagcccgccgg
agaaagagagcccgatgtgcacgctgaaccactccatcagcaggaacaccagccccgcca
ggacggcgtgaatgccatacagcacgggtgcaacaaacaggaaggagaactcaatcggct

cggtgataccgcagacgatggaagtgatcgcgccagagagcataatgcccttcacacgcg
ctatgttttccgggctggcgcagcggtagatcgccagcgcaatcgccggaatacagaaca
tcttgatcggcgtcagcccggccataaaggttccggcggtaatcggcacctgatctttca
actgggcaaagaagataagctggtcgccatgcaccacctgcccggctttgttcacgtact
cgccaaactgcagccagaacgtcggccaccagacatggttaagaccaaacgggatcagca
gccgctcgacaaaaccgaacaggaaggcggacacgcccggcccctgcaccgtcatggtat
tcgacaggccattaatcagatgctgaaccggcggccagaccaccgccatcaccagcccga
caaacagcgcggcgaaggaggtcactatcggcacaaatcgcttgccggagaagaactcca
gccatgaaggaagctggatgcggtaatagcgtttatacagccaggcggcgataatcccga
taatgatgccgccaaacacgccggtctgcagcgaagggatgcccagcaccatcgtgtagt
cacgcacctgcgccaccgattcgggcgtgatccccagaaactggccgatggtgacgttca
taatcaaaaacccggcgatcgccgacagcccggcaatgccctgatcgtcactaagtccca
ccgccacgccgaccgcaaacagcagcggcaggttggcgaaaatcgcactgcccgcttccg
ccatcagcttcagcacgtgaaccagcccgtcagtgccgagaaacggcaggctggcgacaa
tattgggatcctgaaagctaacaccaaaagccagcaaaataccggcggcaggcagcatgg
caatggggatcattaacgatcgcccgatgctttgtaacatttgcagaaggttgaatcttt
tcatctcgatctcctgagcgcgtctttatgacagggattcgcacaacagcaggttctgct
ccgaacaccttcagtatccgaacccgaaatgattaaaaaaacagcaaccaaagacgaaag
ttatttccggttttcgcttaaaaccggaaaatttatgagggcaatcacgccagataccgt
cagaaagggataaaagaggaaatcgcgttcggcgggaggccatcatatgagtatcttcga
ggcgcattttcgcaggctgcacgcccgctacggcgcaggtcagacgcacgaattacagat
gcaggagatcgccgccatcttcggctgttcggtgcgtaattgtcgtattgcgctaaaaaa
gatgcatcaggaaaaatggctcgactggcagccccagcgcgggcgcggcaagcgctcacg
gctccatctgttaacctcgccggaaaagctgttcagccagaacgtcaataagctgctgga
gaagcaggattacggcaacgtgctgcggtttatcggcaacgacaagtatctgctggatcg
cctgagcctgtggcgctttggggtacaggataaaagcagcgaaacgcgggtacgcatccc
ctactatcgcaatctggatccgcttaacccactcgtcccctgcggcggaccgaacgcca
ccttctgcgccagtgcctgagcggactgacgcgctatgacgccgttcagggcaggatcgt
ccccgatatcgcccactactggacccataaggcactgttgcaaatagtcggtggtgataa
acttatcatccccttttgctgatggagctgcacatgaacccattcaaaggccggcatttt
cagcgtgacatcattctgtgggccgtacgctggtactgcaaatacggcatcagttaccgt
gagctgcaggagatgctggctgaacgcggagtgaatgtcgatcactccacgatttaccgc
tgggttcagcgttatgcgcctgaaatggaaaaacggctgcgctggtactggcgtaaccct

tccgatctttgcccgtggcacatggatgaaacctacgtgaaggtcaatggccgctgggcg

tatctgtaccgggccgtcgacagccggggccgcactgtcgatttttatctctcctcccgt

cgtaacagcaaagctgcataccggtttctgggtaaaatcctcaacaacgtgaagaagtgg

cagatcccgcgattcatcaacacggataaagcgcccgcctatggtcgcgcgcttgctctg

ctcaaacgcgaaggccggtgcccgtctgacgttgaacaccgacagattaagtaccggaac

aacgtgattgaatgcgatcatggcaaactgaaacggataatcggcgccacgctgggattt

aaatccatgaagacggcttacgccaccatcaaaggtattgaggtgatgcgtgcactacgc

aaaggccaggcctcagcattttattatggtgatcccctgggcgaaatgcgcctggtaagc

agagtttttgaaatgtaaggcctttgaataagacaaaaggctgcctcatcgctaactttg

caacagtgccggtaaatccatgctggccctgcaactggccgcacagattgcaggcgggcc

ggatctgctggaggtgggcgaactgcccaccggcccggtgatctacctgcccgccgaaga

cccgcccaccgccattcatcaccgcctgcacgcccttggggcgcacctcagcgccgagga

acggcaagccgtggctgacggcctgctgatccagccgctgatcggcagcctgcccaacat

catggccccggagtggttcgacggcctcaagcgcgccgccgagggccgccgcctgatggt

gctggacacgctgcgccggttccacatcgaggaagaaaacgccagcggccccatggccca

ggtcatcggtcgcatggaggccatcgccgccgataccgggtgctctatcgtgttcctgca

ccatgccagcaagggcgcggccatgatgggcgcaggcgaccagcagcaggccagccgggg

cagctcggtactggtcgataacatccgctggcagtcctacctgtcgagcatgaccagcgc

cgaggccgaggaatggggtgtggacgacgaccagcgccggttcttcgtccgcttcggtgt

gagcaaggccaactatggcgcaccgttcgctgatcggtggttcaggcggcatgacggcgg

ggtgctcaagcccgccgtgctggagaggcagcgcaagagcaagggggtgccccgtggtga

agcctaagaacaagcacagcctcagccacgtccggcacgacccggcgcactgtctggccc

ccggcctgttccgtgccctcaagcggggcgagcgcaagcgcagcaagctggacgtgacgt

atgactacggcgacggcaagcggatcgagttcagcggcccggagccgctgggcgctgatg

atctgcgcatcctgcaagggctggtggccatggctgggcctaatggcctagtgcttggcc

cggaacccaagaccgaaggcggacggcagctccggctgttcctggaacccaagtgggagg

ccgtcaccgctgatgccatggtggtcaaaggtagctatcgggcgctggcaaaggaaatcg

gggcagaggtcgatagtggtggggcgctcaagcacatacaggactgcatcgagcgccttt

ggaaggtatccatcatcgcccagaatggccgcaagcggcaggggtttcggctgctgtcgg

agtacgccagcgacgaggcggacgggcgcctgtacgtggccctgaaccccttgatcgcgc

aggccgtcatgggtggcggccagcatgtgcgcatcagcatggacgaggtgcgggcgctgg

acagcgaaaccgcccgcctgctgcaccagcggctgtgtggctggatcgaccccggcaaaa

ccggcaaggcttccatagataccttgtgcggctatgtctggccgtcagaggccagtggtt

cgaccatgcgcaagcgccgccagcgggtgcgcgaggcgttgccggagctggtcgcgctgg

gctggacggtaaccgagttcgcggcgggcaagtacgacatcacccggcccaaggcggcag

gctgacccccccactctattgtaaacaagacatttttatcttttatattcaatggctta

ttttcctgctaattggtaataccatgaaaaataccatgctcagaaaaggcttaacaatat

tttgaaaaattgcctactgagcgctgccgcacagctccataggccgctttcctggctttg

cttccagatgtatgctattctgctcctgcagctaatggatcaccgcaaacaggttactcg

cctggggattccctttcgacccgagcatccgtatgagactcatgctcgattattattatt

atagaagcccccatgaataaatcgctcatcattttcggcatcgtcaacataacctcggac

agtttctccgatggaggccggtatctggcgccagacgcagccattgcgcaggcgcgtaag

ctgatggccgagggggcagatgtgatcgacctcggtccggcatccagcaaccccgacgcc

gcgcctgtttcgtccgacacagaaatcgagcgtatcgcgccggtgctggacgcgctcaag

gcagatggcattcccgtctcgctcgacagttatcaacccgcgacgcaagcctatgccttg

tcgcgtggtgtggcctatctcaatgatattcgcggttttccagacgctgcgttctatccg

caattggcgaaatcatctgccaaactcgtcgttatgcattcggtgcaagacgggcaggca

gatcggcgcgaggcacccgctggcgacatcatggatcacattgcggcgttctttgacgcg

cgcatcgcggcgctgacgggtgccggtatcaaacgcaaccgccttgtccttgatcccggc

atggggtttttctgggggctgctcccgaaacctcgctctcggtgctggcgcggttcgat

gaattgcggctgcgcttcgatttgccggtgcttctgtctgtttcgcgcaaatcctttctg

cgcgcgctcacaggccgtggtccgggggatgtcggggccgcgacactcgctgcagagctt

gccgccgccgcaggtggagctgacttcatccgcacacacgagccgcgcccccttgcgcgac

gggctggcggtattggcggcgctaaaagaaaccgcaagaattcgttaactgcacattcgg

gatatttctctatattcgcgcttcatcagaaaactgaaggaacctccattgaatcgaact

aatatttttttggtgaatcgcattctgactggttgcctgtcagaggcggagaatctggt

gattttgtttttcgacgtggtgacgggcatgccttcgcgaaaatcgcacctgcttcccgc

cgcggtgagctcgctggagagcgtgaccgcctcatttggctcaaaggtcgaggtgtggct

tgccccgaggtcatcaactggcaggaggaacaggagggtgcatgcttggtgataacggca

attccgggagtaccggcggctgatctgtctggagcggatttgctcaaagcgtggccgtca

atggggcagcaacttggcgctgttcacagcctatcggttgatcaatgtccgtttgagcgc

aggctgtcgcgaatgttcggacgcgccgttgatgtggtgtcccgcaatgccgtcaatccc

gacttcttaccggacgaggacaagagtacgccgcagctcgatcttttggctcgtgtcgaa

cgagagctaccggtgcggctcgaccaagagcgcaccgatatggttgtttgccatggtgat

ccctgcatgccgaacttcatggtggaccctaaaactcttcaatgcacgggtctgatcgac

cttgggcggctcggaacagcagatcgctatgccgatttggcactcatgattgctaacgcc

gaagagaactgggcagcgccagatgaagcagagcgcgccttcgctgtcctattcaatgta

ttggggatcgaagcccccgaccgcgaacgccttgccttctatctgcgattggaccctctg

acttggggttgatgttcatgccgcctgtttttcctgctcattggcacgtttcgcaacctg

ttctcattgcggacaccttttccagcctcgtttggaaagtttcattgccagacgggactc

ctgcaatcgtcaagggattgaaacctatagaagacattgctgatgaactgcgcggggccg

actatctggtatggcgcaatgggaggggagcagtccggttgctcggtcgtgagaacaatc

tgatgttgctcgaatatgccggggagcgaatgctctctcacatcgttgccgagcacggcg

actaccaggcgaccgaaattgcagcggaactaatggcgaagctgtatgccgcatctgagg

aacccctgccttctgcccttctcccgatccgggatcgctttgcagctttgtttcagcggg

cgcgcgatgatcaaaacgcaggttgtcaaactgactacgtccacgcggcgattatagccg

atcaaatgatgagcaatgcctcggaactgcgtgggctacatggcgatctgcatcatgaaa

acatcatgttctccagtcgcggctggctggtgatagatcccgtcggtctggtcggtgaag

tgggctttggcgccgccaatatgttctacgatccggctgacagagacgacctttgtctcg

atcctagacgcattgcacagatggcggacgcattctctcgtgcgctggacgtcgatccgc

gtcgcctgctcgaccaggcgtacgcttatgggtgcctttccgcagcttggaacgcggatg

gagaagaggagcaacgcgatctagctatcgcggccgcgatcaagcaggtgcgacagacgt

catactagatatcaagggcactgttgcaaagttagcgatgaggcagccttttgtcttatt

caaaggccttacatttcaaaaactctgcttaccaggcgcatttcgcccaggggatcacca

taataaaatgctgaggcctggcctttgcgtagtgcacgcatcacctcaatacctttgatg

gtggcgtaagccgtcttcatggatttaaatcccagcgtggcgccgattatccgtttcagt

ttgccatgatcgcattcaatcacgttgttccggtacttaatctgtcggtgttcaacgtca

gacgggcaccggccttcgcgtttgagcagagcaagcgcgcgaccataggcgggcgcttta

tccgtgttgatgaatcgcgggatctgccacttcttcacgttgttgaggattttacccaga

aaccggtatgcagctttgctgttacgacgggaggagagataaaaatcgacagtgcggccc

cggctgtcgacggcccggtacagatacgcccagcggccattgaccttcacgtaggtttca

tccatgtgccacgggcaaagatcggaagggttacgccagtaccagcgcagccgtttttcc

atttcaggcgcataacgctgaacccagcggtaaatcgtggagtgatcgacattcactccg

cgttcagccagcatctcctgcagctcacggtaactgatgccgtatttgcagtaccagcgt

acggcccacagaatgatgtcacgctgaaaatgccggcctttgaatgggttcatgtgcagc

tccatcagcaaaaggggatgataagtttatcaccaccgactatttgcaacagtgccatat

caagcgacttctcctatcccctgggaacacatcaatctcaccggagaatatcgctggcca

aagccttagcgtaggattccgccccttcccgcaaacgaccccaaacaggaaacgcagctg

aaacgggaagctcaacacccactgacgcatgggttgttcaggcagtacttcatcaaccag

caaggcggcactttcggccatccgccgcgccccacagctcgggcagaaaccgcgacgctt

acagctgaaagcgaccaggtgctcggcgtggcaagactcgcagcgaacccgtagaaagcc

atgctccagccgcccgcattggagaaattcttcaaattcccgttgcacatagcccggcaa

ttcctttccctgctctgccataaggggtctgacgctcagtggaacgaaaactcacgttaa

gggattttggtcatgagattatcaaaaaggatcttcacctagatccttttaaattaaaaa

tgaagttttaaatcaatctaaagtatatatgagtaaacttggtctgacagttaccaatgc

ttaatcagtgaggcacctatctcagcgatctgtctatttcgttcatccatagttgcctga

ctccccgtcgtgtagataactacgatacgggagggcttaccatctggccccagtgctgca

atgataccgcgagacccacgctcaccggctccagatttatcagcaataaaccagccagcc

ggaagggccgagcgcagaagtggtcctgcaactttatccgcctccatccagtctattaat

tgttgccgggaagctagagtaagtagttcgccagttaatagtttgcgcaacgttgttgcc

attgctgcaggcatcgtggtgtcacgctcgtcgtttggtatggcttcattcagctccggt

tcccaacgatcaaggcgagttacatgatcccccatgttgtgcaaaaaagcggttagctcc

ttcggtcctccgatcgttgtcagaagtaagttggcagcagtgttatcactcatggttatg

gcagcactgcataattctcttactgtcatgccatccgtaagatgcttttctgtgactggt

gagtactcaaccaagtcattctgagaatagtgtatgcggcgaccgagttgctcttgcccg

gcgtcaacacgggataataccgcaccacatagcagaactttaaaagtgctcatcattgga

aaacgttcttcggggcgaaaactctcaaggatcttaccgctgttgagatccagttcgatg

taacccactcgtgcacccaactgatcttcagcatcttttactttcaccagcgtttctggg

tgagcaaaaacaggaaggcaaaatgccgcaaaaaagggaataagggcgacacgaaaatgt

tgaatactcatactcttcctttttcaatattattgaagcatttaccagggttattgtctc

atgagcggatacatatttgaatgtatttagaaaaataaacaaataggggttccgcgcaca

tttccccgaaaagtgccacctgacgtctaagaaaccattattatcatgacattaacctat

aaaaataggcgtatcacgaggccctttcgtcttcaagaattttataaaccgtggagcggg

caatactgagctgatgagcaatttccgttgcaccagtgcccttctgatgaagcgtcagca

cgacgttcctgtccacggtacgcctgcggccaaatttgattcctttcagctttgcttcct

gtcggccctcattcgtgcgttctaggatcctccggcgttcagcctgtgccacagccgaca

ggatggtgaccaccatttgccccatatcaccgtcggtactgatcccgtcatcaatgaacc

ggactgccacgccctgagcgtcaaattcctttatcagttggatcatatcggcggcactgt

tgcaaatagtcggtggtgataaacttatcatccccttttgctgatggagctgcacatgaa

cccattcaaaggccggcattttcagcgtgacatcattctgtgggccgtacgctggtactg

caaatacggcatcagttaccgtgagctgcaggagatgctggctgaacgcggagtgaatgt

cgatcactccacgatttaccgctgggttcagcgttatgcgcctgaaatggaaaaacggct

gcgctggtactggcgtaacccttccgatctttgcccgtggcacatggatgaaacctacgt

gaaggtcaatggccgctgggcgtatctgtaccgggccgtcgacagccggggccgcactgt

cgatttttatctctcctcccgtcgtaacagcaaagctgcataccggtttctgggtaaaat

cctcaacaacgtgaagaagtggcagatcccgcgattcatcaacacggataaagcgcccgc

ctatggtcgcgcgcttgctctgctcaaacgcgaaggccggtgcccgtctgacgttgaaca

ccgacagattaagtaccggaacaacgtgattgaatgcgatcatggcaaactgaaacggat

aatcggcgccacgctgggatttaaatccatgaagacggcttacgccaccatcaaaggtat

tgaggtgatgcgtgcactacgcaaaggccaggcctcagcattttattatggtgatcccct

gggcgaaatgcgcctggtaagcagagtttttgaaatgtaa
